# Probing Skin Barrier Recovery on Molecular Level Following Acute Wounds: An In Vivo/Ex Vivo Study on Pigs

**DOI:** 10.3390/biomedicines9040360

**Published:** 2021-03-31

**Authors:** Enamul Haque Mojumdar, Lone Bruhn Madsen, Henri Hansson, Ida Taavoniku, Klaus Kristensen, Christina Persson, Anna Karin Morén, Rajmund Mokso, Artur Schmidtchen, Tautgirdas Ruzgas, Johan Engblom

**Affiliations:** 1 Department of Biomedical Science, Faculty of Health and Society, Malmö University, SE-205 06 Malmö, Sweden; tautgirdas.ruzgas@mau.se (T.R.); johan.engblom@mau.se (J.E.); 2 Biofilms-Research Center for Biointerfaces (BRCB), Malmö University, SE-205 06 Malmö, Sweden; 3 Timeline Bioresearch AB, Scheelevägen 2, SE-223 63 Lund, Sweden; lbm@timelinebioresearch.se (L.B.M.); it@timelinebioresearch.se (I.T.); kk@timelinebioresearch.se (K.K.); 4 Galenica AB, Medeon Science Park, SE-205 12 Malmö, Sweden; Henri.Hansson@galenica.se (H.H.); AnnaKarin.Moren@galenica.se (A.K.M.); 5 Department of Occupational and Environmental Dermatology, Lund University, Skåne University Hospital, SE-205 02 Malmö, Sweden; Christina.ML.Persson@skane.se; 6 Department of Solid Mechanics & MAX IV Laboratory, Lund University, SE-221 00 Lund, Sweden; rajmund.mokso@maxiv.lu.se; 7 Division of Dermatology and Venereology, Department of Clinical Sciences, Lund University, SE-221 84 Lund, Sweden; artur.schmidtchen@med.lu.se; 8 Copenhagen Wound Healing Center, Bispebjerg Hospital, Department of Biomedical Sciences, University of Copenhagen, DK-2400 Copenhagen, Denmark

**Keywords:** skin barrier, stratum corneum, lipid, acute wound, in vivo/ex vivo, trans-epidermal water loss (TEWL), pH, histology, polarization transfer solid state NMR (PTssNMR), small and wide-angle X-ray diffraction (SWAXD)

## Abstract

Proper skin barrier function is paramount for our survival, and, suffering injury, there is an acute need to restore the lost barrier and prevent development of a chronic wound. We hypothesize that rapid wound closure is more important than immediate perfection of the barrier, whereas specific treatment may facilitate perfection. The aim of the current project was therefore to evaluate the quality of restored tissue down to the molecular level. We used Göttingen minipigs with a multi-technique approach correlating wound healing progression in vivo over three weeks, monitored by classical methods (e.g., histology, trans-epidermal water loss (TEWL), pH) and subsequent physicochemical characterization of barrier recovery (i.e., small and wide-angle X-ray diffraction (SWAXD), polarization transfer solid-state NMR (PTssNMR), dynamic vapor sorption (DVS), Fourier transform infrared (FTIR)), providing a unique insight into molecular aspects of healing. We conclude that although acute wounds sealed within two weeks as expected, molecular investigation of stratum corneum (SC) revealed a poorly developed keratin organization and deviations in lipid lamellae formation. A higher lipid fluidity was also observed in regenerated tissue. This may have been due to incomplete lipid conversion during barrier recovery as glycosphingolipids, normally not present in SC, were indicated by infrared FTIR spectroscopy. Evidently, a molecular approach to skin barrier recovery could be a valuable tool in future development of products targeting wound healing.

## 1. Introduction

**Figure 1:**
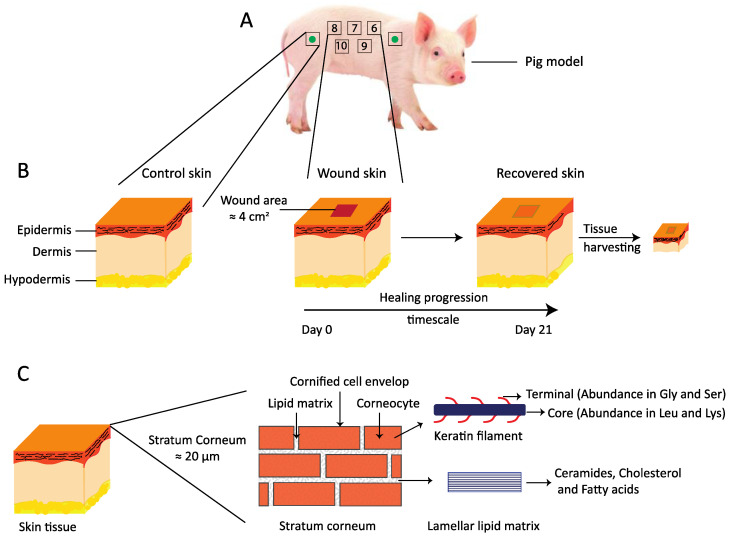
(**A**) A schematic of the wound healing study design. The study was performed on two non-naïve Göttingen minipigs. A total of 10 wounds were generated on each pig, five on the left side of the pigs back and five on the right side, as shown here with numbers from 6 to 10. The green filled circle inside the square indicates regions from where the control skin tissues were harvested at the end of the in vivo study. (**B**) A simplified drawing showing three major layers of the skin: the epidermis, the dermis, and the hypodermis. The wound incision area, which is approximately 2 × 2 cm^2^, is shown, along with the time scale for healing progression over 21 days. After the in vivo study, the recovered skin was harvested for further evaluation ex vivo. (**C**) A cartoon presenting the thin top layer of the epidermis, the stratum corneum, which can be illustrated with bricks and mortar. The bricks in the cartoon represent anucleated corneocytes, mainly composed of keratin filaments. The corneocytes are embedded in a lipid lamellar matrix constituting the mortar. These lipid lamellae mainly comprise ceramides, cholesterol, and free fatty acids.

Skin is one of the largest organs in terrestrial life, comprising an essential barrier towards the external harsh environment and being paramount for our survival. It has numerous vital functions, where perhaps the most important is to maintain body homeostasis, and among others preventing excessive water loss [1,2,3]. Mammalian skin consists of three layers: the epidermis, dermis, and hypodermis (Figure 1) [4]. The epidermis can be further subdivided into four distinct layers: stratum basale (SB), stratum spinosum (SS), stratum granulosum (SG), and stratum corneum (SC). Skin barrier function is assured by the outermost layer of the epidermis—the SC [1,5]. Formation of the barrier begins in the deepest layer of the epidermis where keratinocytes in SB proliferate and gradually migrate through SS and SG [6,7]. During their migration, the cells flatten, anucleate, and adopt the typical size and shape of corneocytes when they reach SC, and finally they are expelled from the skin surface by desquamation. In normal skin, the epidermis undergoes constant renewal with an average turnover of 20 to 30 days [8].

The SC is generally about 10–15 µm thick and consists of a lipid–protein complex where 10–15 layers of protein-bound stacked dead cells, corneocytes, are embedded in a multilamellar lipid matrix [9,10]. The corneocytes are filled with keratin intermediate filaments, proteins (enzymes), and compounds constituting the natural moisturizing factor (NMF), and weigh approximately 85% of the dry SC [11,12]. Corneocytes are responsible for mechanical properties such as viscoelasticity and plasticity of the SC [13]. The extracellular lipids in SC are derived from lamellar bodies found in SG as membrane-bound granules and are responsible for skin barrier function. During the transition from SG to SC, the lamellar bodies secrete their lipid content (phospholipids, glucosylceramides, sphingomyelin, and cholesterol) together with hydrolytic enzymes to the extracellular space through exocytosis [9,14]. These lipids are then further enzymatically processed to the barrier constituting lipids (mainly ceramides (Cer), cholesterol (Chol), and free fatty acids (Ffa)), which together form stacks of structured solid lipid lamellae in the extracellular space of SC [15,16]. In healthy human SC, the lipids exhibit both a long (LPP) and a short (SPP) periodicity phase with repeat distances of approximately 13 and 6 nm, respectively, while the lipid chains pack laterally predominantly in an orthorhombic arrangement [17,18]. The mainly solid lipid lamellae also comprise a small fraction of fluid lipids that play an important role for the macroscopic barrier properties [19,20]. In several skin diseases, e.g., atopic dermatitis and psoriasis, the barrier function of the skin is compromised [21,22,23,24]. The impaired barrier function is often attributed to alterations in SC lipid composition and their lamellar assembly [25]. The fraction of long acyl Cers is, e.g., significantly reduced in the diseased skin, and these specific lipids are key to the formation of LPP, which plays a crucial role in the SC barrier function [26,27]. It has furthermore been reported that lipids in diseased skin preferentially pack in a less dense hexagonal instead of orthorhombic lateral arrangement [26,28].

The SC is often conceptualized by the “brick and mortar” model in which the corneocytes are the bricks and the intercellular lipids serve as the mortar [29]. Unlike cell/plasma membranes, the lipids in SC are mainly solid at ambient temperature and thus form a robust barrier [29,30]. Nevertheless, despite the solid nature of the extracellular lipid matrix and the very low permeability of SC, the barrier is not completely sealed. A minute imperceptible fraction of water (about 300–400 mL/day) evaporates continuously from the skin surface, commonly referred to as trans-epidermal water loss (TEWL) [31]. Injury or other damage to the skin perturb the barrier functionality and require immediate action for rapid wound closure, healing, and restoration. The wound healing process is highly complex and involves a series of subsequent overlapping phases: coagulation, inflammation, proliferation, and remodeling [32]. In acute wounds, the healing normally progresses through these different phases, but the cascade pathway is not always smooth and can come to a halt, as seen with chronic wounds [33,34]. Lack of proper diagnostics and mistreatment often results in scar formation, delayed healing, and recurrent wounds.

There are several factors that may promote wound healing, where hydration and pH in particular are claimed to play a crucial role [35,36,37,38,39,40,41]. Superfluous or reduced hydration may impede healing by, e.g., precluding formation of low molecular weight anti-inflammatory compounds, essential for the process [42]. One example is amino acids in the skin, sourced through proteolytic degradation of filaggrin, which requires hydrated conditions [43]. It has furthermore been demonstrated that wounds with alkaline pH have a lower healing progression rate [44,45]. Under normal circumstances, the skin surface is acidic as a result of, e.g., secreted Ffas from sebaceous glands, NMFs, and other filaggrin degradation products from the keratinocytes [46,47,48,49,50,51,52,53]. Skin’s acidic nature and associated pH gradient is disturbed in wounds, and the more alkaline pH favors protease activity, destroying the cellular matrix and the growth factors that are essential for wound healing [40,54]. Therefore, restoring an acidic milieu in the wound bed would effectively control infection, protease activity, oxygen circulation, etc., which are essential for faster healing rate [55,56]. Several studies have indeed shown the benefits of acidic supplementation in treating burn wounds and skin infections, as well as reducing bacterial colony formation on the skin surface [40,57,58].

Fully recovered skin with no loss of skin appendages should possess a barrier functionality close to that of healthy control skin. However, this recovery may not be straightforward when substantial tissue damage occurs, leading to wide open wounds. Our body’s natural response to such acute trauma would be to reestablish the skin barrier function and maintain body homeostasis, i.e., prevent excessive water loss [59,60]. We hypothesize that rapid wound closure is more important than immediate perfection of the barrier, although it is reasonable to assume that various types of treatment may also facilitate perfection and thereby minimize, e.g., degree of scar formation. As a first step we address this question by monitoring the natural wound healing progression and skin barrier recovery on pigs in vivo. Determining the quality of the restored cutaneous tissue down to molecular level will provide further understanding on how successful the healing has been and serve as a control in future testing of potential treatment to improve healing rate and barrier perfection. We decided to use minipigs as the test model since pig skin is anatomically and physiologically very similar to human skin [61]. Both humans and pigs share similar epidermal turnover time, heal through physiologically similar processes, and also have almost identical skin barrier constitution. These similarities make pigs popular test subjects when it comes to probe pharmacokinetics, skin barrier properties, wound healing progression, etc., and serve as a strong argument for our selection here.

More specifically, the aim of the current paper was to gain further understanding on the mechanism of wound healing on molecular level by studying the natural healing progression of acute wounds in vivo and then evaluate the quality of the restored tissue in comparison to healthy control skin from the same individuals. Two pigs were employed in parallel, and wound healing was followed over three weeks by visual inspection, monitoring degree of wound closure, pH at wound site, skin surface temperature, and TEWL. The recovered tissue was then excised from the wound sites together with adjacent healthy control skin and subjected to classical histology to examine potential morphological differences. The harvested tissue was also analyzed with physicochemical methods to gain further details on molecular structure and dynamics of the SC and its constituents. Using small and wide-angle X-ray diffraction (SWAXD) and Fourier transform infrared (FTIR) spectroscopy, we obtained information regarding lipid lamellae and protein organization in the SC. Dynamic vapor sorption (DVS) provided water uptake capacity of SC with respect to ambient humidity, and polarization transfer solid state NMR (PTssNMR) provided further details on the dynamics of fluid and solid material in the SC that could be linked to the SC organization probed by SWAXD. This multi-technique approach correlating wound healing progression monitored by classical established methods and subsequent physicochemical characterization of barrier recovery provides a unique insight into molecular aspects of healing. We are confident that it would serve as a very valuable support for future considerations in the development of new supplements that could further speed up and improve the healing by, e.g., reducing scar formation.

## 2. Materials and Methods

### 2.1. Materials

Bovine pancreas trypsin and ethanol were purchased from Sigma-Aldrich Chemie GmbH, Schnelldorf, Germany. K_2_SO_4_, which was used to prepare saturated salt solution to maintain constant humidity in the desiccator, was obtained from Merck, Darmstadt, Germany. Water used to prepare salt solutions was of Millipore quality produced by MilliQ water filtration system from Merck, Darmstadt, Germany with a resistivity of 18 MΩ.cm at 25 °C.

### 2.2. In Vivo Study

#### 2.2.1. Animal Model

The in vivo wound healing study was performed on 2 castrated male Göttingen minipigs from Ellegaard Göttingen Minipigs, Denmark. The minipigs were non-naïve and aged between 9 and 10 months.

#### 2.2.2. Ethical Permission

The in vivo wound healing study was conducted according to the Swedish ethical conduct of animal experiments with license number M131–16 and extension 974/2019.

#### 2.2.3. Wound Induction

On day 0, the pigs were anesthetized with an intramuscular injection of Zoletile mixture (Tiletamine, Zolazepam, Butorphanol, Ketamine, Xylazine) 1 mL/10 kg. When needed, additional Zoletil mixture was given in a reduced dosage (0.5 mL/10 kg). The pigs were also continuously given oxygen via a face mask. Eye ointment (Viscotears 2 mg/g, Bausch + Lomb GmbH, Berlin, Germany) was applied over the cornea to avoid desiccation. Heart rate and oxygen saturation of blood (SpO_2_) were continuously monitored with a pulse oximeter. The back of the pigs was clipped with an electric clipper, washed with a sponge containing antimicrobial skin cleaner based on chlorhexidine gluconate (Medi-scrub, Rovers medical devices B.V., Lekstraat, the Netherlands), shaved with a disposable razorblade, and wiped with chlorhexidine (Klorhexidinsprit 5 mg/mL, Fresenius Kabi AB, Uppsala, Sweden). The wound areas were marked out with a marker-pen and subsequently disinfected with 70% ethanol (Solveco, Ref 1393, Stockholm, Sweden).

Five full thickness wounds were induced on each side of both pigs, yielding a total of 10 wounds per pig. Each wound was approximately 2 × 2 cm^2^, and there was 4 cm in between each wound. The wounds were induced with a sterile disposable scalpel (Paragon, size 22, Ref P508, Swann Morton Ltd., Sheffield, United Kingdom) in a depth of approximately 4–8 mm. The skin in the wounded area was fixed with forceps and removed from the subcutaneous fat with scissors.

In order to avoid the wounds drying out, we used a sterile gauze moist with 0.9% NaCl to cover the wounds until all wounds had been induced.

#### 2.2.4. Pain Treatment

During anesthesia on day 0, the pigs were given an intramuscular injection with buprenorphine (Temgesic 0.3 mg/mL, Indivior UK Limited, East Yorkshire, UK) as well as a fentanyl plaster (Fentanyl ratiopharm 50 μg/h, Ratiopharm GmbH, Ulm, Germany) attached to the abdominal skin. The fentanyl plaster was estimated to continuously provide analgesia for approximately 4 days. Additionally, pigs were administered oral paracetamol (Alvedon 500 mg, GlaxoSmithKline Consumer Healthcare AB, Brondby, Denmark) the first 2 days after wound induction. At the dressing change on day 4, the fentanyl dose was reduced by changing to another fentanyl plaster (Fentanyl Sandoz 25 µg/h, Sandoz A/S, Copenhagen, Denmark), and following this period, no further treatment was considered necessary.

#### 2.2.5. Dressing Change

Primary layer: Each wound was individually covered with an absorbent foam dressing (Mepilex Transfer, ref 294800, Mölnlycke Health Care, Sweden) cut into pieces of 3 × 3 cm^2^, and an 5 × 5 cm^2^ adhesive film (Tegaderm Roll, Ref 16002, 3 M Health Care, Dusseldorf, Germany) to avoid leakage.

Secondary layer: Sterile gauze (Cutisoft Cotton, ref 71738-17, BSN medical, Hamburg, Germany) attached with sports tape (Strappal, Ref 71490-00, BSN Medical, Vibraye, France) was used to cover the wounded area and the primary dressing.

Tertiary layer: An elastic, water-repellent bandage (Vet–Flex, Kruuse, Langeskov, Denmark) was bandaged around the pigs neck, back, and belly.

#### 2.2.6. Study Termination

Study termination was conducted at study day 21.

The pigs were anesthetized (Zoletil mixture). Blood samples, measurements, and photographs were taken before the pig was euthanized with an intravenous overdose of pentobarbital (Exagon vet. 400 mg/mL, Richter Pharma AG, Wels, Austria). Following euthanasia, the wounds were sampled and frozen for subsequent analysis.

### 2.3. Trans-Epidermal Water Loss (TEWL) Measurement

TEWL was evaluated using a Delfin VapoMeter wireless with serial number SWL4001. The VapoMeter is equipped with a closed cylindrical chamber that contains sensors for humidity and temperature. The TEWL is calculated from the linearly increased humidity in the chamber after placing the device in contact with the skin. The evaporation rate follows Fick’s Law of diffusion, indicating the quantity being transported per a defined area and period of time. The results were expressed in grams per square meter per hour (g/m^2^·h). All the TEWL values were recorded at ambient surrounding conditions, which were controlled externally to approximately 50% relative humidity (RH) and at 23 °C.

### 2.4. Measurement of pH and Skin Temperature

The pH of the wounds and control skin as well as skin surface temperature were measured using the SOFT PLUS (Callegari S.r.I., Parma, Italy) instrument equipped with pH electrode and temperature probe. The pH was recorded in selected wound and control positions on every occasion of dressing change during the healing cycle. The temperature was recorded only on the control skin surface at those occasions when we also measured pH and TEWL.

### 2.5. Wound Closure Monitoring and Photographic Documentation

After each removal of the primary dressings, photographs were taken with a digital camera (Nikon D50, lens: Sigma 50 mm 1:2.8 DG Macro D) of each wound position to document wound closure. Each photograph included a label of pig number and wound number, as well as a ruler for size determination. The wound areas were measured using the program Image J Fiji.

### 2.6. Histology

Frozen skin tissues from control and healed skin at position 10 were used to prepare histology slides. Classical hematoxylin and eosin (H&E) staining was performed, followed by epoxy embedding using the standard protocol as described by Arcega et al. [62]. The stained tissues were cut to a thickness of 5 µm slices and imaged with a light microscope (Olympus BX 43 with 40× objective lens) using the software ImageView version x64.

### 2.7. Sample Treatment Ex Vivo

Skin for ex vivo experiments were harvested from all the healed skin positions (approximately 3 × 3 cm^2^) as well as from the control skin sites (approximately 5 × 5 cm^2^) close to the head and tail region of the pigs’ backs (see Figure 1A) after the in vivo session and stored at −80 °C until further used. For histology preparations, we used the excised tissue without further modifications. For other experiments where isolated SC was required, we carefully removed SC from the remaining skin according to the protocol described below.

### 2.8. Isolation of Stratum Corneum (SC)

Prior to SC separation, the excised skin was thawed and carefully rinsed under cold tap water with the epidermis side upwards to avoid any contact of subcutaneous fat with the epidermis. Then, the hair was removed from the skin surface with a trimmer. The control skin was dermatomed (Dermatome, Integra LifeSciences, Plainsboro, NJ, USA) to a thickness of ≈500 μm, while the smaller pieces of healed skin had to be dissected using a scalpel. The skin was then placed on a filter paper soaked in trypsin solution (0.2 wt % trypsin in MilliQ) and kept at 4 °C for about 20 h. The SC sheets were peeled off from the viable epidermis using forceps and rinsed in excess MilliQ water 5 times to get rid of all the trypsin. The SC sheets were dried under vacuum in a desiccator and stored at −20 °C for subsequent use.

### 2.9. Sample Preparation Method for SWAXD, NMR, DVS, and FTIR Studies

To prepare samples for NMR experiments, we used approximately 20 mg of dry SC, which was then equilibrated for 48 h at 32 °C either at dry conditions or at 97% RH, provided by a saturated K_2_SO_4_ salt solution in the desiccator. After equilibration, the samples were quickly transferred to NMR inserts (Bruker) with screw caps to avoid dehydration/hydration and subsequently placed in the NMR rotors (Bruker) with caps on it. The same method but with less sample (3–5 mg) was used to prepare samples for SWAXD experiments. After equilibration, the samples were quickly transferred to screw-tight sandwich cells with polyethylene film as support windows for SWAXD measurements. The DVS and the attenuated total reflectance (ATR)–FTIR measurements were performed at dry conditions only, using approximately 5–7 mg and 1–3 mg of dry SC, respectively.

### 2.10. Small and Wide Angle X-ray Diffraction (SWAXD)

SWAXD studies were performed using a SAXSLab Ganesha 300XL instrument (SAXSLAB ApS, Skovlunde, Denmark), equipped with 2D 300K Pilatus detector (Dectris Ltd., Baden, Switzerland). The scattering intensity (I) was recorded as a function of the scattering vector q in reciprocal Ångström (Å^−1^), where q is defined as $q\;=\;\frac{4\pi sin\theta }{\lambda }$. Here, θ is the scattering angle and λ is the wavelength of the incident X-ray beam, where *λ*(Cu–K_α_) = 1.54 Å. The *d*-spacing was calculated from the peak position of q by using equation $d\;=\;\frac{2\pi }{q}$. The two-dimensional (2D) scattering pattern recorded by the detector was radially averaged using the software SAXSGui to obtain 1D I vs. q data. The exposure time varied from 15 to 120 min and the temperature was controlled to 32 °C using an external circulating water bath.

### 2.11. Polarization Transfer Solid State Nuclear Magnetic Resonance (PTssNMR)

PTssNMR is a combination of three different experiments that are performed on the same sample in a sequential manner, i.e., direct polarization (DP), cross polarization (CP), and insensitive nuclei enhanced by polarization transfer (INEPT) [63,64]. The experiments were carried out on a Bruker Avance AVII 500 NMR spectrometer equipped with a 4 mm CP/magic angle spinning (MAS) probe, operated at 5 kHz frequency, with ^1^H and ^13^C resonance frequencies of 500 and 125 MHz, respectively. The temperature calibration was performed with methanol and set to 32 °C in all experiments [65]. A spectral width of 250 parts per million (ppm) was used and the number of scans per experiment was 2048 with an acquisition time and a recycle delay of 0.05 and 5 s, respectively. This gave a total estimated time of approximately 9 h for all 3 experiments. Data processing was done using a line broadening of 20 Hz, zero filling from 1597 to 8192 time domain points, Fourier transformation, phase correction, and baseline correction using an in-house Matlab code partially derived from matNMR [66,67].

### 2.12. Dynamic Vapor Sorption (DVS) Measurements

Water sorption measurements were performed using the TA Instruments (New Castle, DE, USA) Q5000 SA Dynamic vapor sorption microbalance (DVS). To compare the sorption isotherms, we ran both control and healed SC samples in parallel in a single experiment. Dry samples were placed on 2 separate pans hanging on a balance in the DVS and exposed to a stream of N_2_ with controlled RH at 32 °C. The water sorption of the samples was continuously recorded by the microbalance.

### 2.13. Fourier Transform Infrared (FTIR) Spectroscopy

ATR–FTIR spectra of control and healed SC samples were recorded using a Thermo Nicolet Nexus 6700 instrument from Thermo Scientific (Waltham, MA, USA). All the experiments were performed in the attenuated total reflectance (ATR) mode in dry conditions and at ambient temperature.

## 3. Results

In the present work we employed Göttingen minipigs aged 9 to 10 months to study healing progression of acute wounds in vivo and to compare the quality of the restored tissue down to a molecular level with that of control healthy skin excised from the same individuals. A comparative analysis in terms of skin barrier recovery at the molecular level concerning SC molecular dynamics, lipid composition, and lipid–protein structural organization was made with the long-term aim of obtaining a better understanding of the mechanism behind wound healing and how it can be affected to promote and perfectionate tissue recovery. Ten full thickness wounds, five on each side of the pigs back, were incised on each pig on day 0 when initiating the in vivo study (Figure 1A). The study was carried out over 21 days, which was a fairly reasonable time to recover the skin tissue with all its major layers (Figure 1B). During the in vivo cycle, we measured a set of physical parameters, i.e., TEWL, pH, and skin surface temperature, in order to evaluate the healing progression in real time and compared with control skin. The wounds were also photographed and monitored visually for any sort of inflammation that might delay the healing process. When the in vivo part was terminated on day 21, healed skin was harvested from all wound sites on each pig together with adjacent control healthy skin. The different layers in the healed tissue were visualized along with that of control skin using hematoxylin and eosin. The molecular details regarding SC lipid and protein organization (Figure 1C) were obtained with SWAXD, PTssNMR, and FTIR. All results are presented and discussed below. First, we present the data obtained in vivo in real time during wound healing progression, and then the data obtained on ex vivo post-healing excised tissue are described.

### 3.1. Wound Healing Progression In Vivo

#### 3.1.1. Visualizing Wound Closure and Healing Cycle In Vivo

**Figure 2:**
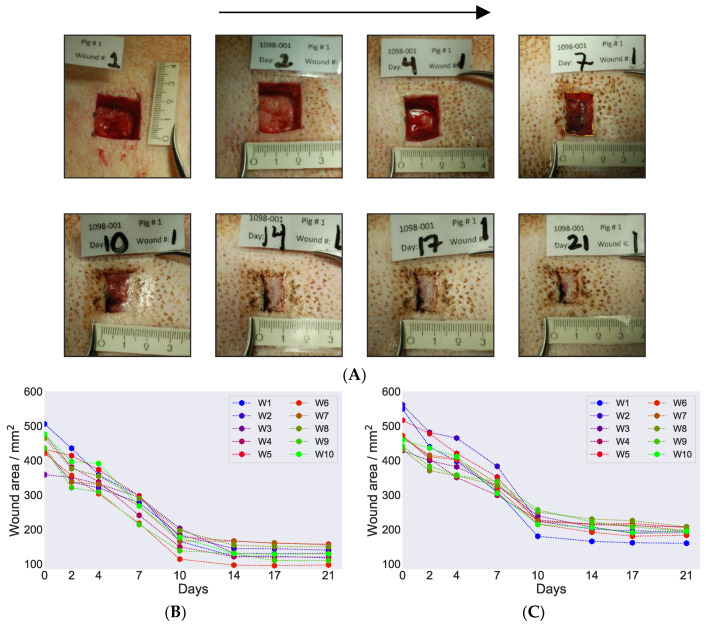
(**A**) Representative wound images on pig 1 at various time points during the healing progression cycle. The arrow on top of the figure indicates ascending date for healing cycle and the readers are encouraged to follow the images in the arrow direction. The images presented origin from position 1 and were captured on the days when the dressing was changed. The name plates on top of the images indicate the in vivo wound study number, pig number, study days when the wound dressing was changed, and the wound position number. The ruler on the bottom of the images was used as a guide to set the scale when calculating the wound marked area, e.g., see marking on day 7. Calculated wound area was presented as a function of days during the healing cycle for pig 1 (**B**) and pig 2 (**C**). The labelling W1, W2 and so on in the figure legends indicate the various wound positions on each pig.

Images were captured for all wound positions on both pigs throughout the healing cycle from day 0 of wound incision until day 21 when the in vivo part of the study was terminated. Figure 2A displays representative images for pig 1 on wound position 1 and provides a qualitative overview of the healing progression. Two more representative image series of the healing cycle on the two pigs are provided in Figures S1 and S2. The images clearly demonstrate an overall good skin recovery at the end of the healing cycle on day 21. When we examined more closely, formation of granulation tissue on the wound bed on day 7 was noticeable, and on day 10, further contraction from the side was also evident. This indicates that both primary and secondary wound healing took place and full wound closure was observed on day 14. A similar wound closure on day 14 was observed for all the wounds on both pigs on day 14. No sign of infection was observed during the healing process when the dressings were changed in either of the pigs. From day 14 and further until day 21, no visual differences were noticed.

When comparing the healing progression cycle on pig 1 and pig 2, we noticed a few differences. At the beginning, the overall healing moved faster on pig 2. However, at the later part of the cycle, from day 14 onwards, the healing process was faster on pig 1. The wounds were narrower in pig 1 compared to pig 2 on day 14, which indicates faster tissue regeneration and contraction from the sides in pig 1.

We further calculated wound area closure of each wound on both pigs during the healing cycle at various time points. Table 1 tabulated wound area values calculated for wound position 1. For all other wound positions, the calculated area values are provided in Table S1. The detailed procedure of area calculation is given in the supplementary text. Figure 2B,C shows the calculated wound area for all wounds plotted as a function of time during the healing cycle for pig 1 and pig 2. The overall trend looked similar in both pigs, with the area gradually decreasing during the first 10 days and plateauing after day 10 until the end of the healing cycle on day 21. This is expected when examining wound images and it can be seen that wounds were almost sealed on day 10. The remaining minor sealing happened before day 14, for which we saw minor decrease in the closure of the wounds. On day 17 post-wound induction, the wounds were completely closed. The wound area calculations therefore provided the values of the wound scar lines and indicated no change in those markings during the healing process. Comparing pig 1 and pig 2, the area revealing wound closure with marked scar formation was higher in pig 1 compare to pig 2 (Figure 2B,C).

#### 3.1.2. TEWL and pH as Barrier Indicator of Healed Skin

**Figure 3:**
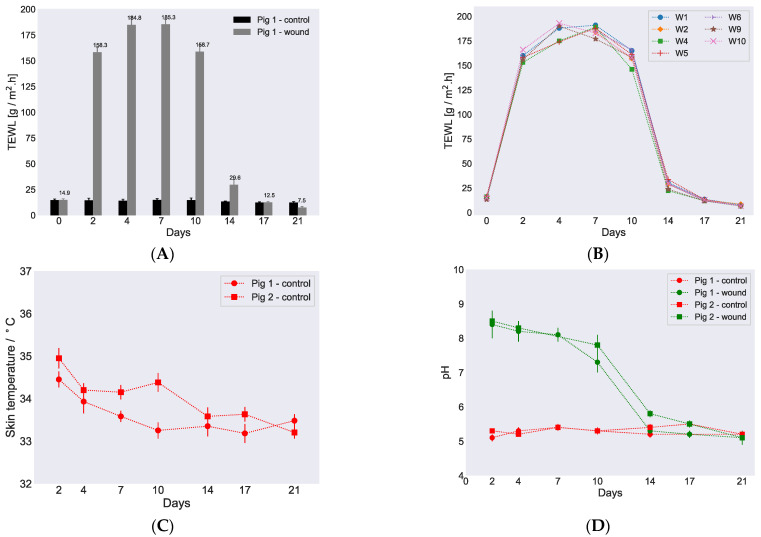
Variations in TEWL, skin temperature, and pH during healing. (**A**) Measured TEWL vs. time for control skin and healing skin for pig 1, position 1. On day 0, the readings were recorded before the wound incision. (**B**) TEWL values vs. time for individual wounds obtained from pig 1 when the wound dressings were changed. (**C**) Skin surface temperature vs. time measured on both pigs. (**D**) pH measurements on the wounds (position 1, pig 1, and position 5, pig 2) and corresponding control sites were plotted vs. time for both pigs. All the TEWL and pH values were recorded at ambient controlled conditions, i.e., approximately 50% relative humidity (RH) and 23 °C. Error bars depict standard deviation.

There always exists a water gradient in the skin with lower water activity on the skin surface, regulated by the water activity of the ambient, and a higher water activity in the deeper layers of the dermis. This gradient in water activity gives rise to diffusional transport of water from the inside of the body towards the ambient and subsequent evaporation from the skin surface. This continuous imperceptible water loss is normally referred to as TEWL and is widely measured and used as an indicator to assess the skin barrier function. In general, a higher water loss from the skin surface would indicate a compromised/reduced skin barrier and vice versa. We here recorded TEWL values on various wounds in both pigs and also on the control site at various time points during the wound healing study in order to assess skin barrier recovery. Figure 3A presents bar plots of the recorded TEWL values (average with standard deviation) against days for control and healed skin sites at various time points of the healing cycle for pig 1. Additionally, the individual values for several wounds on pig 1 are plotted as a function of time in Figure 3B. The corresponding curves for pig 2 are provided in Figure S3. Table 1 also compiles representative TEWL values for wound position 1 in both pigs along with control measurement values. All the recorded TEWL values from the various wound positions in both pigs at different days of the wound healing as well as all the control measurements during the in vivo study are tabulated in Table S2.

An overall similar trend for the recorded TEWL values was observed for both control and healing skin in both pigs as shown in Figure 3A,B. The average TEWL value recorded on day 0 for control skin was approximately 15 ± 0.2 for pig 1 and 14 ± 0.1 g/m^2^·h for pig 2. TEWL values were also recorded on day 0 for the particular skin sites before wound incision. On every occasion, when the wound dressings were changed and TEWL was recorded for healing skin, we also measured TEWL for control skin. The recorded control values were very similar for both pigs throughout the study period. When TEWL was measured for healing skin on day 2, the recorded values were, as expected due to the open wound sites and moist environment, high, i.e., 149.2 ± 1.3 and 153.2 ± 1.5 g/m^2^·h for pig 1 and pig 2, respectively. Higher TEWL values were also recorded on day 4 in both pigs and decreased only slightly on day 7 and further on day 10. The highest TEWL value was recorded for pig 2 on day 4 and was 191 ± 1.6 g/m^2^·h. On day 14, a dramatic decrease in TEWL was observed in both pigs with recorded values of 32 ± 0.3 and 34 ± 0.4 g/m^2^·h for pig 1 and pig 2, respectively. The timing of the sharp decrease in TEWL around day 14 was in accordance with wound closure that was also observed on day 14 (Figure 2A, Figures S1 and S2) and established the connection that the measured TEWL values were reduced due to sealing of the wound surface. The TEWL values continued to decrease further and returned to comparable control values on day 17. At this point, the TEWL values recorded were 13 ± 0.4 and 14 ± 0.5 g/m^2^·h for pig 1 and pig 2, respectively. Surprisingly, the TEWL values further reduced on day 21 down to 7.8 ± 0.3 g/m^2^·h in pig 2, which was below the normal values recorded for control skin.

In addition to the determination of TEWL, skin surface temperature and pH of the wounds were also measured during the in vivo study at each occasion where the wound dressings were changed. The recorded skin surface temperature on both pigs at control positions were plotted as a function of study days and presented in Figure 3C, and the values are listed in Table 1. A full list of temperatures recorded at various control positions is revealed in Table S3. The skin surface temperature on both pigs showed similar trends and remained steady with little fluctuations of 1–2 degrees throughout the study.

The pH values measured for two randomly chosen wounds and corresponding control sites are presented in Figure 3D. Hence, pH was determined for wound position 1 for pig 1 and position 5 for pig 2, and the values are revealed in Table 1. All pH values measured for the individual wounds during the healing cycle are provided in Table S4. The average measured pH on the control skin site was 5.3 ± 0.1 for both pigs and was steady during the whole cycle. When the pH values were recorded for the different wounds, a slightly basic pH of around 8.0 was observed in both pigs on day 2 and remained high until day 7. On day 2, the pH values measured were 8.4 ± 0.4 and 8.5 ± 0.3 for pig 1 and pig 2 in wound positions 1 and 5, respectively. The pH of the wounds started decreasing after day 7, and on day 10, the recorded values were 7.3 ± 0.3 and 7.8 ± 0.6 for pig 1 and pig 2 on wound positions 1 and 5, respectively. On day 17, when the wounds were almost dry, the measured pH returned to around 5.5 in both pigs and did not change further during the remaining time of the in vivo cycle, hence resembling the values for the control measurements.

### 3.2. Ex Vivo Characterization of Recovered Tissue after Healing Compared to Control Skin

#### 3.2.1. Morphology of Healed vs. Control Skin

**Figure 4:**
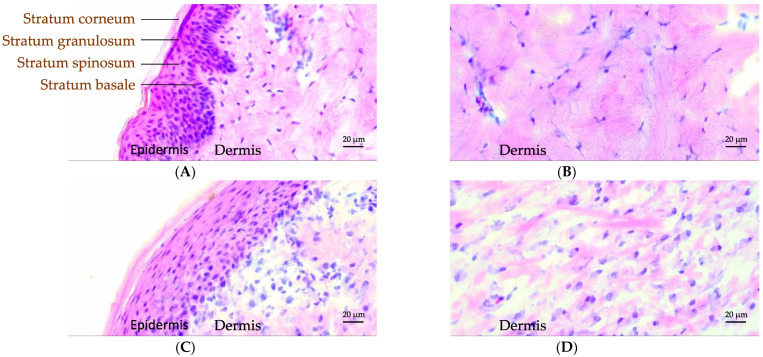
Classical hematoxylin and eosin (H&E) staining of control (**A**,**B**) and healed (**C**,**D**) pig skin. The skin surface is directed towards the top/left. The individual epidermal layers can be identified in the control skin labelled (**A**). Magnifications of the dermis part for control and healed skin are provided in (**B**,**D**).

To evaluate the quality of recovered tissue after healing, we visualized various skin layers with light microscopy using hematoxylin and eosin (H&E) staining. Figure 4 displays images of histological preparations from excised skin of pig 2 at wound position 10 on day 0 (Figure 4A,B) as well as skin harvested after in vivo termination on day 21 (Figure 4C,D). Additional images of control and healed skin tissue are provided in Figure S4. The images captured from skin harvested on day 0 could serve as a control since the healed skin tissue was harvested from the same wound position after terminating the in vivo study.

Figure 4A clearly demonstrates the dermis and the individual layers of the epidermis for control skin as labelled in the image. The corresponding layers were likewise observed in the healed skin, as shown in Figure 4C. However, further details in various layers of the epidermis revealed differences between control and healed skin. In control skin, the dermal epidermal ridges developed from the basal cells were quite prominent, while the healed skin did not present such ridges (Figure 4A,C). The cells in the basal layer of the epidermis in control skin were columnar shaped, ordered, and densely packed (Figure 4A). In the healed skin, on the other hand, these cells were flat, distorted, and disorganized (Figure 4C). As the basal cells undergo progressive maturation during migration towards stratum spinosum and granulosum layers, referred to as keratinization, they became flatter in shape. The healed skin revealed less densely packed cells in epidermis, and the cells were also highly stretched and flattened. The surface layer of the epidermis, the stratum corneum (SC), appeared to have a similar thickness in both control and healed skin (Figure 4A,C). Further morphological differences in the maturation of corneocyte cells in the healed SC could not be discerned from these light microscopy images due to limitations in resolution. Figure 4B,D displays a closeup on the dermis region for both control and healed skin. From these images, we clearly noticed more epithelization in the control compared to healed skin, indicating less developed dermis in recovered skin.

#### 3.2.2. SC Lipid and Protein Molecular Organization Investigated Using SWAXD

**Figure 5:**
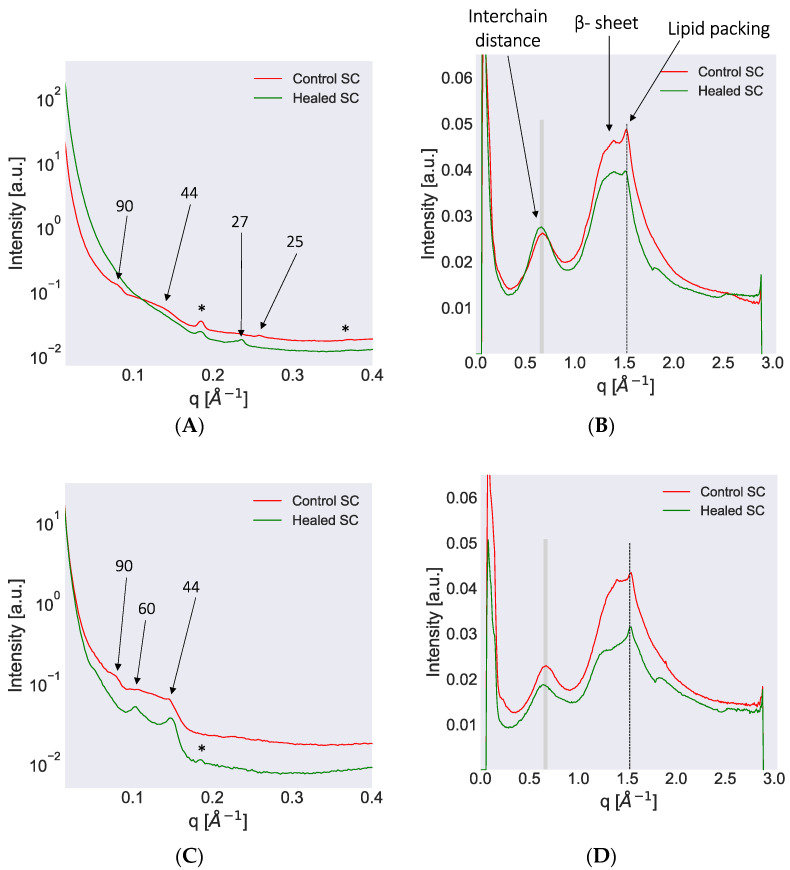
Small and wide-angle X-ray diffraction (SWAXD) spectra of control and healed stratum corneum (SC) excised from pig 1 and recorded at dry (**A**,**B**) and wet (97% RH) conditions (**C**,**D**). The SAXD spectra are provided on the left column whereas the WAXD spectra are on the right. In the SAXD region, several peaks attributed to lipid lamellar ordering and keratin packing are identified. The numbers with arrows indicate the *d*-spacing in Å for various peaks. Phase-separated crystalline Chol was also detected in some spectra and then indicated by an asterisk (*) sign. In the WAXD region, the shaded lines indicate peaks originating from the keratin interchain distance and show a change in peak position following hydration of the sample. The lipid acyl chain ordering indicated by dotted lines do not show a shift in peak position when comparing dry and hydrated conditions. The secondary β-sheet structure of keratin could also be detected and is marked in the plot. All SWAXD measurements were performed at 32 °C, which represents average physiological skin temperature.

SWAXD can provide detailed information on molecular ordering (within approximately 3–150 Å) in the complex architecture of the SC, particularly regarding variations in lipid lamellar organization and lateral hydrocarbon chain packing, protein secondary structure, and keratin filament organization. Figure 5 displays the SAXD and WAXD spectra of the control and healed SC for pig 1 measured at dry conditions and at 97% RH. The corresponding spectra obtained for pig 2 are provided in Figure S5. All the SWAXD peaks detected in SC from the two pigs at dry and wet conditions are compiled in Table 2 along with peaks reported in the literature for pig SC. The SWAXD pattern of both control and healed SC revealed several peaks corresponding to structures formed by SC lipids and protein (Figure 5). A few of these peaks, highlighted in gray in Table 2, showed differences between control and healed SC, while the remaining peaks did not change. Below, we present the origin of specific peaks and then describe the differences we observed between control and healed SC.

SAXD pattern from control SC at dry condition showed a weak peak at *q* ≈ 0.07 Å^−1^, which corresponded to a *d*-spacing of approximately 90 Å (Figure 5A). The origin of this peak has been interpreted as being due to the keratin filament rod diameter or due to the thickness of corneocyte bound lipids [15,68]. A broad hump centered around *q* ≈ 0.14 Å^−1^ that corresponded to a *d*-spacing of approximately 44 Å was also observed in control SC at dry conditions (Figure 5A). This hump may be the second order of the 90 Å peak and/or the third order of the LPP, which has also been reported in the literature [15,68,69,70]. Additionally, another peak at *q* ≈ 0.25 Å^−1^, which corresponded to a *d*-spacing of approximately 25 Å, was detected and could be the second order of a SPP with shorter *d*-spacing (Figure 5A). The dry SC of control skin also revealed a clear peak at *q* ≈ 0.18 Å^−1^, which corresponded to a *d*-spacing of approximately 33 Å (Figure 5A). This peak originated from crystalline Chol present in the samples [15]. When hydrating the control sample at 97% RH, the peaks at *q* ≈ 0.07 and 0.14 Å^−1^, which corresponded to *d*-spacings of 90 and 44 Å, respectively, could still be detected along with the Chol peak at *q* ≈ 0.18 Å^−1^ (Figure 5C). In addition, in the hydrated state, another peak at *q* ≈ 0.1 appeared, which corresponded to a *d*-spacing of approximately 60 Å (Figure 5C). This peak could be attributed to the first order of the SPP in accordance with the literature [15]. When examining the SAXD pattern of the healed SC at dry conditions, we observed a peak at *q* ≈ 0.23 Å^−1^ corresponding to a *d*-spacing of approximately 27 Å (Figure 5A). No other peaks were observed at dry conditions except the Chol peak at *q* ≈ 0.18 Å^−1^. Healed SC at wet conditions revealed two peaks at *q* ≈ 0.1 and 0.14 Å^−1^, which corresponded to *d*-spacings of approximately 60 and 44 Å (Figure 5C). The 60 Å peak could be attributed to the first order of the SPP, whereas the 44 Å peak might have been due to keratin rod diameter or third order of LPP, as described for control SC. The SAXD pattern for pig 2 showed similar trends as observed for pig 1 in both control and healed SC (Figure S5A,C).

The WAXD spectrum of control SC at dry conditions revealed several prominent peaks. The peak at *q* ≈ 0.67 Å^−1^, which corresponded to a *d*-spacing of approximately 9.3 Å is attributed to the keratin interchain distance of two-polypeptide chains (Figure 5B) [15,68,71,72]. Hydration at 97% RH led to a shift in this peak position to *q* ≈ 0.65 Å^−1^, which corresponded to an increase in *d*-spacing to approximately 9.7 Å (Figure 5D). At dry conditions, the control SC also exhibited a peak at approximately 1.39 Å^−1^, which corresponded to a *d*-spacing of 4.5 Å (Figure 5B). Previously, this peak was attributed to the β-sheet secondary structure of keratin [73,74]. The control SC furthermore showed a prominent peak at *q* ≈ 1.52 Å^−1^ at dry conditions, which corresponded to a *d*-spacing of approximately 4.1 Å (Figure 5B). This *d*-spacing is typical of hexagonally packed lipid acyl chains [15]. This peak did not shift on hydration. The WAXD spectrum of the healed SC exhibited similar features as the control SC. The keratin interchain distance in healed SC showed a shift in peak position from *q* ≈ 0.67 Å^−1^ in dry state to 0.64 Å^−1^ in wet state, which corresponded to a change in *d*-spacing from approximately 9.3 to 9.8 Å (Figure 5D). The protein secondary structure was less pronounced in healed SC at wet conditions and the lipid chain packing was not affected by the hydration. The WAXD pattern in pig 2 showed similar trends as observed for pig 1 at dry and wet conditions for both control and healed SC (Figure S5B,D).

In summary, using SWAXD, we identified differences in the protein molecular structure between control and healed SC (keratin filament rod diameter and interchain distances). Only small differences were resolved for the lipid organization, which might have been due to the relatively low flux in the inhouse X-ray source.

#### 3.2.3. Water Uptake in SC Examined by DVS and ^1^H NMR

**Figure 6:**
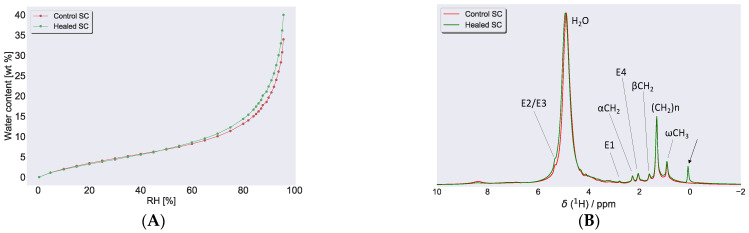
(**A**) Water sorption measurements for control and healed SC at 32 °C, expressed as water content, (in wt % with respect to dry SC) plotted as a function of RH. (**B**) ^1^H NMR spectra recorded on control and healed SC at 97% RH. The peak close to 5 ppm in both spectra was due to water in the sample from hydration at 97% RH. Several sharp peaks attributed to lipid molecular segments (assignments in Figure 7E) were also detected, indicative of fluid lipids present in both samples. The arrow close to 0 ppm in the healed SC was due to the silicon present in the wound dressing.

Water sorption isotherms provided a relation between the water content of a sample and the ambient relative humidity (RH). Figure 6A depicts the sorption isotherm of control and healed SC. When sorption measurements were performed with DVS to examine water uptake in SC from both control and healed SC, the spectra were almost identical in the low humidity regime (<50% RH, Figure 6A). In the higher humidity regime (>50% RH), the water uptake was slightly higher in healed SC compared to control SC. At 96% RH, the water uptake in healed SC was approximately 40 wt %, whereas control SC took around 33 wt %. Further evaluation of water uptake in these samples were performed with ^1^H NMR, and Figure 6B shows NMR spectra of control and healed SC equilibrated at 97% RH. Both ^1^H NMR spectra for the respective control and healed SC showed a pronounced peak at approximately 4.75 ppm, indicative of water present in the samples.

#### 3.2.4. Molecular Structure and Dynamics of SC Constituents Examined by PTssNMR

PTssNMR relies on the natural abundance of ^13^C present in the sample and provides details on the SC lipid and protein mobility with close to atomic resolution. A detailed description of the method and its applicability is given elsewhere [11,76,77,78,79]. Briefly, the PTssNMR method comprises three individual experiments (i.e., DP, CP, and INEPT) performed on the very same sample. It relies on the natural abundance of ^13^C in the sample. The DP experiment utilizes a direct pulse and acquires all ^13^C signals present in the sample and can be used as a reference. The CP and INEPT experiments involve polarization transfer from ^1^H⟶^13^C, where CP boosts signals for solid/rigid carbons and INEPT does so for mobile/fluid carbons present in the sample [63,64]. When overlaying the DP, CP, and INEPT spectra in one plot, one can unveil a detailed molecular picture regarding the SC molecular environment.

**Figure 7:**
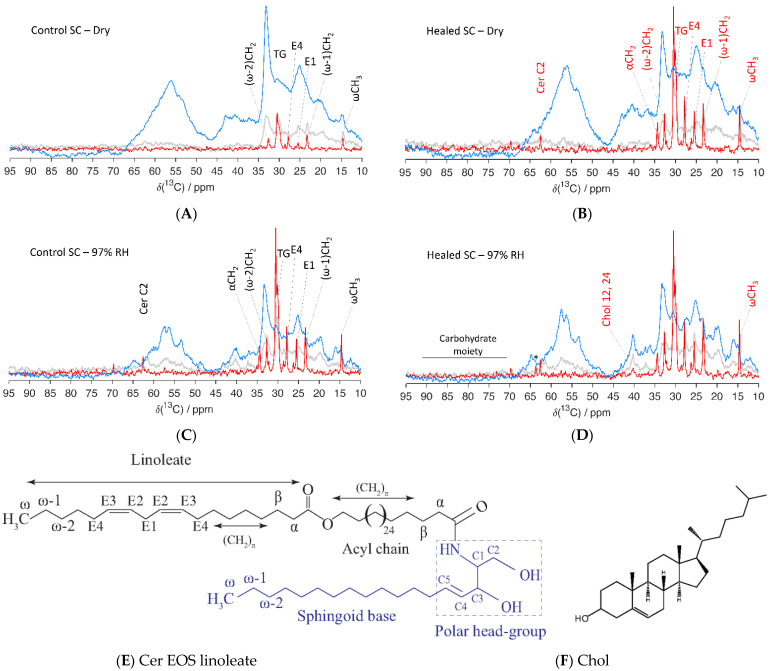
Natural abundance ^13^C polarization transfer solid state NMR (PTssNMR) study on control (**A**,**C**) and healed (**B**,**D**) SC samples in dry (**A**,**B**) and wet (**C**,**D**) conditions. The individual direct polarization (DP) (grey), cross polarization (CP) (blue), and insensitive nuclei enhanced by polarization transfer (INEPT) (red) spectra were overlaid in all experiments for the purpose of comparison. The resonance lines originating from various SC lipid molecular segments along with Chol are labelled in the spectra. The labelling is provided in black for control and red in healed SC when changes in the INEPT signals were observed compared to control SC. The peak labeled with (*) in the healed SC at 97% RH might indicate the presence of glycosphingolipid. Assignments of various lipid molecular segments with their numberings are depicted with a representative ceramide (Cer) structure—Cer EOS linoleate (**E**), as well as for Chol (**F**).

Here, PTssNMR spectra were recorded for control and healed SC equilibrated at both dry conditions and at 97% RH. The spectra for the lower chemical shift region (10–95 ppm) are shown in Figure 7, while the corresponding spectra for the higher ppm range (100–150 ppm) are provided in Figure S7. The forest of peaks in all these NMR spectra revealed a complex molecular architecture in the SC. The first impression when looking at the spectra is the dominant CP signal (blue), which demonstrates that the main part of SC is in solid/rigid state in both control and healed SC, independent on the degree of hydration. However, the presence of an INEPT (red) signal is also evident, indicating that a fraction of the SC constituents is in mobile state. The notable lipid peaks detected in all these spectra originated from the lipid acyl chain terminals (ωCH_3_ at ≈14.6 ppm, (ω-1)CH_2_ at ≈23.3 ppm, and (ω-2)CH_2_ at ≈32.5 ppm), lipid main chain methylene (–CH_2_–)_n_ all-trans (AT), and trans/gauche (TG) isomers at ≈33 and 31 ppm. We further noticed molecular mobility in the unsaturated lipid acyl chains. The E1 and E4 carbon segments showed mobility at approximately 25.7 and 27.6 ppm, respectively (assignments in Figure 7E), while the E2 and E3 segments showed mobility in the higher ppm range, at approximately 128 and 131 ppm, respectively (Figure S7). Signals from the lipid headgroups were also observed at approximately 34.8, 56.3, and 62.3 ppm for αCH_2_, Cer C1, and for Cer C2, respectively (assignments in Figure 7E).

In dry state, both control and healed SC showed mobility in the terminal and central parts of the lipid acyl chains. However, the mobility was much higher in healed SC compared to control SC. The unsaturated carbon segments in the chains also showed mobility in both control and healed SC (Figure 7 and Figure S7), and the mobility of E1 and E4 carbon segments was higher in healed SC compared to control SC. Similar higher mobility of E2 and E3 segments were noticed in the high ppm regime for healed SC (Figure S7). Furthermore, the healed SC showed mobility in the headgroup region for αCH_2_ and Cer2 carbon segments, which was not seen with control SC. No molecular mobility could be detected for Chol carbons and protein components for either control or healed SC at dry conditions. When hydrating SC at 97% RH, lipid mobility was increased in all the carbon segments for which we detected signals in dry conditions. We also observe additional mobility in the lipid head-groups at αCH_2_ for control SC, which was not seen in dry state. No differences in mobility between control and healed SC could be detected at hydrated conditions, except for a slightly higher mobility of the lipid chain terminal ωCH_3_ and Chol in healed SC. For healed SC, we observed an additional signal at 63.5 ppm and a few weak signals between 65 and 100 ppm, which could be attributed to the presence of a carbohydrate moiety from glycosphingolipids present in the sample [80,81]. Note, we did not notice any mobility in the protein segments even at 97% RH in either control or healed SC, which differed from previous observations from samples of pig ear SC [75]. The weak signal at approximately 40 ppm could be attributed to the Chol 12,14 carbon segments.

## 4. Discussion

Cutaneous wound healing is a complex process and is not yet fully understood. Hence, in order to investigate the wound healing mechanism and evaluate post-wound healing skin barrier recovery in comparison with control skin barrier, we conducted an in vivo study in Göttingen minipigs. In general, Göttingen minipigs are well-established animal models for human wound healing and therefore form the model of choice for the current study. The length of the in vivo study was 21 days, which was an appropriate time for wound closure. No visual sign of inflammation was observed in any of the induced wound sites during the study, which indicated a normal healing process with no complications involved. The wound healing progression was examined in real time during the in vivo period using traditional approaches, e.g., monitoring wound closure and measuring wound pH and TEWL, which allowed for the comparison of these parameters to those previously reported in literature on wound healing. Histological examination on excised healed skin tissue was also performed to evaluate the recovered dermis and epidermis and correlate with the literature. As our main interest lies in the healed skin barrier recovery in comparison to the control skin barrier, we performed further ex vivo tissue characterization with several physicochemical methods, including SWAXD, FTIR, water uptake capacity, and PTssNMR characterization of the SC, which revealed molecular details and provided insight to what extent the wound has been recovered when compared with control SC. The physicochemical characterization was then further discussed in relation to the traditional measures of wound healing progression performed during the in vivo session.

In control healthy skin, the TEWL, which is commonly used as a measure of the skin barrier efficiency, has been reported to be 10–15 g/m^2^·h [82,83,84]. In the current study, the obtained TEWL value was around 15 g/m^2^·h for the control skin. Differences in measured TEWL could be due to instrumental aspects, and variations in ambient conditions such as humidity, temperature, etc. [85]. When TEWL was measured repeatedly on control skin, the values were very similar in both pigs. The measured TEWL on the wound sites were significantly higher than control at the beginning of the wound healing cycle and dropped abruptly on day 14, which was due to sealing of the wound opening as visualized in the wound images. This shows an excellent correlation between sharply decreased TEWL and wound sealing on day 14. The TEWL of the wounds reached the control values on day 17 of the healing cycle. Interestingly, the TEWL decreased below control values when measured on day 21. This reduction was not due to fluctuations in humidity and temperature, which were controlled and stable during the whole in vivo cycle. The low TEWL in healed skin might instead have been due to possible loss of skin appendages, thicker skin due to scar formation, differences in SC molecular composition, etc.

An important aspect of assessing wound healing progression is to evaluate the pH of the wound fluid. The wound healing proceeds more rapidly at an acidic pH compared to alkaline or neutral pH [44,86]. This has been explained by the fact that protease inhibitors and several other enzymes essential for wound healing work best at slightly acidic conditions [40,56]. When pH was measured on the wound sites, an alkaline pH around 8.0 was recorded at the beginning of the wound healing cycle. Similar high pH values on the wound sites have also previously been reported in the literature and could have several reasons, including exposure of wound tissue to the body’s physiological pH, release of molecules on the wound site as a result of acute wounds, bacterial colonization, or the production of ammonia generated by urease with urea as substrate [56,87]. As the healing progresses and the amount of wound fluid gradually decreases, the wound pH also shows a declining trend. This is indicative of wound healing progression without visual inflammation. It is also consistent with literature reports showing that the wound environment generally progresses from an initial alkaline condition through a neutral and then acidic state during the healing cycle [45,54,88,89,90]. The pH of the control skin was also recorded and shown to be slightly acidic, i.e., around 5.0. We here acknowledge the fundamental problem of interpreting the measured pH reading obtained by placing an electrode on a dry skin surface. In the present wound healing study, the wounds were moist with substantial wound fluid at the beginning of the healing cycle. This leads to a reliable measurement of wound pH until day 10 when the wounds were still moist. On day 14 and onwards, due to the sealing of wounded area, the wound surfaces were dry, and interpreting the pH reading again posed a challenge. However, a pH value around 5.1 was recorded on day 14 and onwards when the wound surfaces were dry, which was in agreement with skin surface pH values reported in the literature [47,48].

Restoration of barrier function to prevent further damage or infection of the wounds is an important aspect of the healing process [41,59]. It is plausible that there is a clear advantage to the wounded individual with respect to accelerate the sealing of the open wound while the remaining healing underneath may proceed at a different rate to perfectionate the barrier. Indeed, histological observations of the wound tissues in both samples revealed several morphological differences in various layers of the epidermis and dermis. In the healed skin tissue, the cells in various layers of the epidermis were still deformed, not densely packed, and organized, which indicated incomplete healing in those layers when compared with control skin. The dermis was furthermore less epithelialized in healed skin when compared to control skin. These observations clearly indicate that after three weeks of wound healing in vivo, cell morphology and tissue rebuilding in the deeper layers had not reached the same level as in the control skin. Due to lack of resolution in light microscopy, details in the superficial thin layer of the skin, the SC, could not be resolved, and instead physicochemical methods were used to detect molecular differences between control and healed skin three weeks post-wound induction in the Göttingen minipigs.

As the transport properties and mechanical strength of SC largely depends on SC hydration, we investigated the water uptake of SC isolated from the excised skin at varying hydrating conditions. When we compared the water uptake of healed and control SC, the sorption profiles looked very similar with only a slight difference at higher RH. The water uptake at 97% RH in both healed and control SC was measured to be between 35 and 40 wt %, which is in line with previous reports [75]. Further examination of SC with SWAXD did, however, reveal differences in protein keratin organization. The control SC exhibited a peak with *d*-spacing 90 Å, which was attributed to the keratin filament rod diameter and/or the thickness of corneocyte-bound lipids. The corresponding peak was absent in the healed SC, which might indicate that the corneocytes did not fully mature and/or that the keratin organization was less developed. Furthermore, in the healed SC, the keratin interchain distance showed more pronounced swelling upon hydration compared to control SC, which could be another indication of less developed keratin. The structural information obtained from SWAXD can be compared to the information on molecular mobility in the keratin filament amino acids. The PTssNMR data showed no mobility in any of the carbon segments of the keratin filament at the humidities investigated (Figure 7). This contrasts from previous reports on SC from pig ears showing that the protein segments become mobile at RH values above a threshold hydration corresponding to ≈85% RH [75]. It is possible that the sample treatment and the SC extraction and washing procedures may lead to excessive loss of polar NMF molecules. Lack of NMF and other small molecules could lead to reduced water uptake in the corneocytes and subsequent reduction in molecular mobility [91]. It is also possible that the SC molecular properties vary slightly between different sites on the body, which also may explain some of the observed differences compared to previous studies.

The molecular organization of SC lipids have been shown to play an important role in the skin barrier function. When we examined lipid lamellar organization using SWAXD and PTssNMR, both control and healed SC revealed a short lamellar phase, i.e., SPP, phase-separated Chol, along with hexagonal lateral packing of the lipid acyl chains. This is in accordance with literature reports for pig SC, although orthorhombic packing of the lipid chains has also been reported in pig SC on the basis of FTIR data [15,92]. No long lamellar phase, i.e., LPP, was detected in either of the samples. However, the q ≈ 1.4 Å^−1^ peak, which corresponds to a *d*-spacing of 45 Å, has been suggested to originate from a third-order refection of the LPP [15,69]. This peak is present in the control SC while absent in the healed SC at dry conditions. Apart from that, no other differences could be observed in lipid organization between control and healed SC due to the lack of higher flux in the in-house X-ray source, and more detailed investigations would require a synchrotron X-ray source.

While SWAXD provides detailed information on ordered structures, it is less powerful to detect small changes in fluid structures. Changes in molecular mobility of SC components are, however, considered crucial to the material properties of SC, including barrier function [93]. The ^13^C PTssNMR spectra from both control and healed SC show mobility in several lipid segments with increasing mobility at increasing hydration. This is also evident from the ^1^H NMR analysis of control and healed SC at dry conditions (Figure S6) where the peaks originating from lipid acyl chain terminals, main chain (CH_2_)_n_, lipid head-groups, and double bond unsaturation are sharp and clearly visible. The higher mobility in the healed SC compared to control SC at dry conditions could indicate differences in lipid composition. The healed SC also reveals several other interesting peaks in the region between 55 and 100 ppm, which may come from, e.g., saccharides and phospholipid head-groups [94]. This could indicate presence of glycosphingolipids in the healed SC, a precursor molecule for barrier lipid synthesis that is normally not present in the SC [95]. Incomplete lipid conversion/production during the three weeks we allowed for skin barrier recovery in the current study could indeed affect the SC lipid organization, and our FTIR data also confirm potential presence of glycosphingolipids in the healed SC (Figure S8). A detailed lipid composition analysis of both healed and control SC would help to resolve this issue and will be the subject for a future study.

The healing progression follows the same trends in both pigs when evaluated on the basis of TEWL, pH, and wound closure. TEWL and pH largely returned to control values within three weeks, indicating good healing progression. However, the deeper layers of epidermis and dermis did not mature to resemble control skin within the three-week period. The extracellular lipids of SC did not produce the LPP, and samples from both pigs also revealed the presence of more fluid lipids. Taken together, this indicates that there was a difference in SC lipid composition between the healed skin and the control skin. These phenomena supported our hypothesis that rapid wound closure is crucial for restoring skin barrier, minimizing fluid loss, and preventing further infection while perfecting the lipid composition considered vital for optimal barrier function and restoring the viable parts of the skin underneath may proceed over a longer time scale. The current study may serve as a baseline, as well as a template, for evaluating future formulations, ability to facilitate wound healing time and quality, and possibly reduce scar formation.

## 5. Conclusions

In the current work, we conducted a study on Göttingen minipigs to gain further understanding on the mechanism of wound healing on molecular level by studying the natural healing progression of acute wounds in vivo and then evaluate the quality and the molecular properties of the excised restored tissue in comparison to healthy control skin from the same individuals. The main findings are as follows:

- Acute open wounds sealed within two weeks after incision. Wound closure monitored visually reflected good regeneration of the tissue at the end of the 21 days in vivo session. Histological evaluation of excised healed skin did, however, reveal morphological differences in various layers of the epidermis and less epithelialization in the dermis when compared with control skin.
- The TEWL of the healing skin reached the value of control skin (≈15 g/m^2^·h) within three weeks from wound incision. The pH was alkaline (≈8.0) at the beginning of the healing cycle and became slightly acidic (≈5.1) at the end of the session. Both TEWL and pH provided an excellent correlation to visually determined wound closure.
- SC water uptake capacity was similar in both control and healed SC with slightly higher water uptake in healed SC compared to control SC at higher humidity.
- SWAXD studies on excised SC showed poorly developed keratin organization in healed SC. The extracellular lipid organization in healed SC also exhibited deviations in lamellar structure compared to control SC.
- PTssNMR revealed the presence of more fluid lipids in healed SC compared to control SC. This could be a result of incomplete lipid conversion during barrier recovery as glycosphingolipids, which were normally not present in SC, were detected by FTIR.

We conclude that although the wounds healed within three weeks, barrier recovery on the molecular level was not complete. Topical supplementation comprising compounds that facilitate lipid maturation and affect subsequent organization in SC may help skin barrier recovery and improve healing with, e.g., reduced scar formation as a result. Evidently a molecular approach to wound healing could be a valuable tool in future product development.

## Figures and Tables

**Table 1:** Summary of calculated wound area, trans-epidermal water loss (TEWL), pH, and skin surface temperature measurements performed during the in vivo wound healing. The measurements were recorded at the time points when the dressings were changed during the healing cycle. * For wound area calculations and TEWL measurements, the values corresponding to wound position 1 are provided. ** For pH, the readings provided correspond to wound position 1 for pig 1 and position 5 for pig 2. The skin surface temperature values presented here originate from control measurements on the left side of the pigs’ backs. The “–” sign indicates that no measurement was performed at that particular occasion.

	Wound Area * (mm^2^)	TEWL * (g/m^2^·h)		pH **		Temperature (°C)
Pig 1/Days	Wound	Control	Wound	Control	Wound	Control
0	505.6	14.4	14.4	–	–	–
2	435.1	11.9	160	5	8.4	34.2
4	358.0	12.8	188	5.3	8.2	34.2
7	297.6	14.7	191	5.3	8.1	33.7
10	165.6	12.1	165	5.2	7.3	33.5
14	122.0	12.9	30.5	5.1	5.3	33.5
17	122.5	12.2	13.5	5.2	5.2	33.3
21	117.2	12.3	7.9	5.2	5.1	33.6
Pig 2/Days						
0	549.3	15.3	11.9	–	–	–
2	439.3	13.7	144	5.3	8.5	34.7
4	397.1	11.8	195	5.2	8.3	34.4
7	310.2	13.6	183	5.4	–	34.3
10	180.2	13.7	167	5.2	7.8	34.1
14	165.4	11.0	25.2	5.3	5.8	33.4
17	161.3	12.4	12.6	5.4	5.5	33.7
21	159.9	12.6	7.2	5.1	5.1	33.1

**Table 2:** Compilation of SWAXD peaks detected in control and healed SC spectra for both pigs at dry and wet conditions. The peaks are presented with their *Q* positions (Å^−1^) and corresponding *d*-spacings (Å) and compared with *d*-spacings reported in the literature. The differences observed between control and healed SC at dry and wet conditions are highlighted in gray. (*)—indicates peaks for which higher diffraction orders are reported in the literature; NR—not resolved; RT—room temperature; Chol—cholesterol. All the SWAXD measurements were carried out at 32 °C.

	Dry			Wet			*d* (Å) in Literature for Pig	Remarks
	Pig 1 *Q* (Å^−1^)	Pig 2 *Q* (Å^−1^)	*d* (Å)	Pig 1 *Q* (Å^−1^)	Pig 2 *Q* (Å^−1^)	*d* (Å)		
**Control SC**	–	–	–	–	–	–	120–132 * [15,69]	LPP with higher orders. Observed at RT and recrystallization from 120 °C.
	0.07	–	90	0.07	0.07	90	90 [15]	Keratin rod diameter/thickness of corneocyte bound lipids.
	–	–	–	0.1; 0.2	–	60	60 [15]	SPP. Detected at RT and skin temperature.
	0.25	–	25	–	0.12; 0.25	50–52; 25	– –	SPP first order with shorter *d*-spacing. SPP second order with shorter *d*-spacing.
	0.14	0.14	44	0.14	0.14	44	45 [15,69]	Possibly second order of 90 Å phase/higher order of LPP/other phase.
	0.18	0.18	33	0.18	0.18	33	34 [15,68]	Anhydrous Chol crystals.
	1.52	1.52	4.1	1.52	1.52	4.1	4.1 [15,69,75]	Crystalline hexagonal packing of hydrocarbon lipid tails.
	0.67	0.67	9.3	0.65	0.65	9.7	9.0–10.4 * [15,69,75]	Keratin interchain distance. Swelling upon hydration.
	NR	NR	–	NR	NR	–	5.1–5.4 * [15,69,75]	α-Helical secondary structure of keratin protein.
	1.39	1.39	4.5	1.39	NR	4.5	4.6–4.9 * [15,75]	β-Sheet secondary structure of keratin protein.
**Healed SC**	–	–	–	–	–	–	120–132 * [15,69]	LPP with higher orders. Observed at RT and recrystallization from 120 °C.
	–	–	–	–	–	–	90 [15]	Keratin rod diameter/thickness of corneocyte bound lipids.
	–	–	–	0.1	–	60	60 [15]	SPP with higher orders. Detected at RT and skin temperature.
	0.23	0.12; 0.23	52–54, 27	–	0.12	52	– –	SPP first order with shorter *d*-spacing. SPP second order with shorter *d*-spacing.
	–	–	–	0.14	0.14	44	45 [15,69]	Possibly second order of 90 Å phase/higher order of LPP/other phase.
	0.18	0.18	33	0.18	–	33	34 [15,69]	Anhydrous Chol crystals.
	1.52	1.52	4.1	1.52	1.52	4.1	4.1 [15,69,75]	Crystalline hexagonal packing of hydrocarbon lipid tails.
	0.67	0.65	9.3–9.7	0.64	0.60	9.8–10.4	9.0–10.4 * [15,69,75]	Keratin interchain distance. Swelling upon hydration. More pronounced in healed SC compared to control SC.
	NR	NR	–	NR	NR	–	5.1–5.4 * [15,69,75]	α-Helical secondary structure of keratin protein.
	NR	1.39	4.5	NR	NR	–	4.6–4.9 * [15,75]	β-Sheet secondary structure of keratin protein (not observed in wet condition).

## Data Availability

Not applicable.

## Supplementary Materials

The following are available online at https://www.mdpi.com/article/10.3390/biomedicines9040360/s1: Supplementary text with figure: cutaneous wound area calculation, Table S1: compilation of wound area calculation, Table S2: compilation of measured TEWL values, Table S3: compilation of recorded skin surface temperature, Table S4: compilation of measured pH values, Figure S1: Representative wound images on pig 1, position 4 during the healing cycle, Figure S2: Representative wound images on pig 2, position 10 during the healing cycle, Figure S3: variation in TEWL readings during the healing cycle, Figure S4: Histology images on control and healed skin, Figure S5: SWAXD spectra of control and healed SC excised from pig 2 in dry and wet condition, Figure S6: ^1^H NMR measurements on control and healed SC in dry condition, Figure S7: ^13^C PTssNMR spectra on control and healed SC in dry and wet conditions for high (100–150) ppm region, Figure S8: FTIR spectra of control and healed SC in dry condition.
