# Supplementary material for: Probing Skin Barrier Recovery on Molecular Level Following Acute Wounds: An In Vivo/Ex Vivo Study on Pigs
Source: Biomedicines. 2021 Mar 31;9(4):360. doi: 10.3390/biomedicines9040360 (PMC8065685; doi:10.3390/biomedicines9040360)
Supplement: Supplementary file 1 [file biomedicines-09-00360-s001.zip › biomedicines-1158623-supplementary.pdf]

Supplementary materials

Probing skin barrier recovery on molecular level following acute wounds: an *in vivo/ex vivo* study on pigs

Enamul Haque Mojumdar [1,2,*], Lone Bruhn Madsen [3], Henri Hansson [4], Ida Taavoniku [3], Klaus Kristensen [3], Christina Persson [5], Anna Karin Morén [4], Rajmund Mokso [6], Artur Schmidtchen [7,8], Tautgirdas Ruzgas [1,2] and Johan Engblom [1,2]

[1] Department of Biomedical Science, Faculty of Health and Society, Malmö University, SE-205 06 Malmö, Sweden; tautgirdas.ruzgas@mau.se (T.R.); johan.engblom@mau.se (J.E.)
[2] Biofilms-Research Center for Biointerfaces (BRCB), Malmö University, SE-205 06 Malmö, Sweden
[3] Timeline Bioresearch AB, Scheelevägen 2, SE–223 63 Lund, Sweden; lbm@timelinebioresearch.se (L.B.M.); it@timelinebioresearch.se (I.T.); kk@timelinebioresearch.se (K.K.)
[4] Galenica AB, Medeon Science Park, SE-205 12 Malmö, Sweden; Henri.Hansson@galenica.se (H.H.); AnnaKarin.Moren@galenica.se (A.K.M.)
[5] Department of Occupational and Environmental Dermatology, Lund University, Skåne University Hospital, SE-205 02 Malmö, Sweden; Christina.ML.Persson@skane.se (C.P.)
[6] Department of Solid Mechanics & MAX IV Laboratory, Lund University, SE-221 00 Lund, Sweden; rajmund.mokso@maxiv.lu.se (R.M.)
[7] Division of Dermatology and Venereology, Department of Clinical Sciences, Lund University, SE-221 84 Lund, Sweden; artur.schmidtchen@med.lu.se (A.S.)
[8] Copenhagen Wound Healing Center, Bispebjerg Hospital, Department of Biomedical Sciences, University of Copenhagen, DK-2400 Copenhagen, Denmark

* Correspondence: enamul.mojumdar@gmail.com (E.H.M.); Tel.: +46–40–665 74 69

Supplementary text with figure. *Cutaneous wound area calculation*

Prior to wound incision, approx. 2x2 cm^2 areas were marked on each side of the pigs back. A total of ten areas have been marked, five on each side of the pigs back and numbered from 1 to 10 to indicate the wound positions. Numbers 1 to 5 are assigned to the left side of the pigs back and 6 to 10 are on the right side of the body. With the aid of a scalpel, the wound incision was performed chronologically in those wound positions. The wound incision procedure was similar for both pigs. Each wound was photographed using a Nikon D50 DSLR camera on the day of wound incision as well as on subsequent days when the wound dressings were changed during the healing cycle. While taking the photograph, a ruler was placed next to the wound in order to show the scale. The captured wound photographs were loaded in the software Image J (https://imagej.nih.gov/ij/). The millimetre (mm) unit was chosen in Image J to define the wound area. Using the straighten tool in Image J, a straight line was dragged on the ruler from 0 to 1 cm and set as 10 mm in the Image J. This procedure has now defined the number of pixels corresponding to 10 mm. By using the freehand selection tool, a precise selection has been made around the wound borders and with that selection active, the wound area was measured using the analyse and measure tool in Image J. The calculated wound area is then given in mm^2. This procedure was used to calculate the change in wound area for all wound positions in both pigs until day 10. On day 14, when the wounds were almost dry and fully sealed, there was no visible opening to mark the wound borders. Here we decided to select the area where we noticed the wound scar lines. This procedure was used from day 14 and onwards until day 21, when the *in vivo* study was terminated. Therefore, the calculated area on day 14 and further on represents not the actual wound closure but rather the scar markings on the wounds.

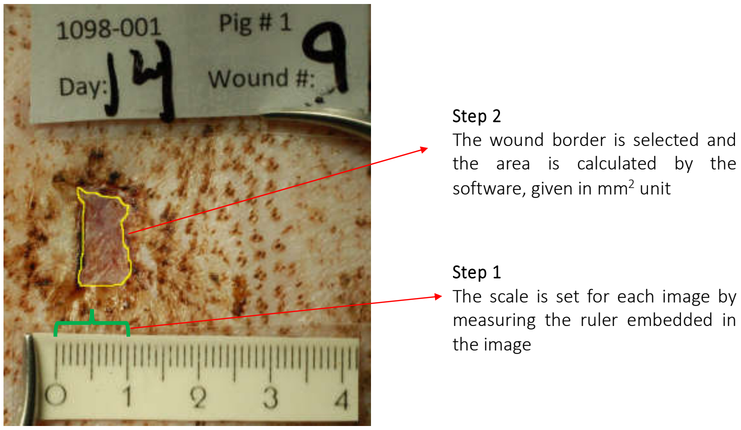

Table S1. Compilation of calculated wound areas at various wound positions for both pigs during the *in vivo* healing cycle.

			Left side – Wound position					Right side – Wound position				
	Days	Date	1	2	3	4	5	6	7	8	9	10
	0	2020–01–27	505.61	428.50	358.50	473.86	431.97	420.30	435.02	464.57	434.37	476.01
	2	2020–01–29	435.11	337.80	351.51	380.47	413.66	355.89	338.01	375.89	321.05	396.61
	4	2020–01–31	358.02	320.58	332.37	338.28	372.89	303.87	331.00	354.92	308.81	390.81
	7	2020–02–03	297.58	277.40	293.59	240.95	297.55	217.77	268.07	290.35	214.23	268.09
Pig 1	10	2020–02–06	165.56	184.20	203.10	149.58	178.85	113.95	166.28	196.78	138.10	177.20
	14	2020–02–10	122.04	144.40	130.80	121.00	166.50	96.40	166.00	154.54	130.00	130.27
	17	2020–02–13	122.46	144.10	129.94	120.00	160.00	95.00	161.00	150.00	110.00	128.00
	21	2020–02–17	117.20	139.85	131.02	120.00	157.22	97.00	155.00	149.50	109.83	129.20
	0	2020–01–27	549.28	561.18	428.29	471.56	516.38	471.29	465.21	431.26	440.61	458.64
	2	2020–01–29	439.34	481.10	399.31	404.14	477.61	410.52	414.63	370.18	382.62	435.63
	4	2020–01–31	397.16	464.67	381.20	351.06	420.13	401.34	405.21	355.77	357.41	411.31
Pig 2	7	2020–02–03	310.26	382.72	327.00	299.18	351.92	317.85	334.68	327.90	339.37	305.03
	10	2020–02–06	180.23	223.79	239.52	227.88	222.12	215.74	225.61	250.57	256.74	214.27
	14	2020–02–10	165.41	201.48	206.98	214.24	215.97	192.15	216.39	229.80	222.25	205.63
	17	2020–02–13	161.38	195.53	189.00	210.32	217.31	180.38	213.11	225.67	207.66	191.05
	21	2020–02–17	159.98	194.56	190.66	207.68	205.34	183.21	196.33	207.36	194.88	194.67

Table S2. Compilation of measured TEWL values at various wound and control positions for both pigs during the *in vivo* wound healing study. The "–" sign indicates that the measurements were not performed at that particular occasion.

			Left side – control position					Right side – control position				
	Days	Date	1	2				3	4			
	0	2020–01–27	14.4	16.0				13.3	15.0			
	2	2020–01–29	11.9	15.5				16.1	12.6			
	4	2020–01–31	12.8	13.8				15.6	16.1			
	7	2020–02–03	14.7	16.2				13.8	–			
	10	2020–02–06	12.1	16.3				15.3	–			
	14	2020–02–10	12.9	13.1				13.8	–			
	17	2020–02–13	12.2	11.6				13.0	–			
	21	2020–02–17	12.3	11.9				13.1	13.8			
Pig 1			Left side – wound position					Right side – wound position				
	Days	Date	1	2	3	4	5	6	7	8	9	10
	0	2020–01–27	14.4	–	–	16	16.2	–	14.4	–	13.3	15
	2	2020–01–29	160	–	–	153	158	–	157	–	156	166
	4	2020–01–31	188	–	189	175	174	–	–	–	190	193
	7	2020–02–03	191	184	–	189	188	185	–	–	177	183
	10	2020–02–06	165	157	–	146	161	166	–	–	158	158
	14	2020–02–10	30.5	28.8	28.4	22.3	33.7	29.2	–	40.1	23.8	29.7
	17	2020–02–13	13.5	12.7	14.2	11.9	13.3	11.9	10.6	12.6	11.8	12.9
	21	2020–02–17	7.9	8.6	6.8	6.6	6.6	6.9	8.8	9.0	7.1	6.6
			Left side – control position					Right side – control position				
	Days	Date	1	2				3	4			
	0	2020–01–27	15.3	14.2				11.9	15.8			
	2	2020–01–29	13.7	15.3				14.1	12.1			
	4	2020–01–31	11.8	12.3				13.8	13.4			
	7	2020–02–03	13.6	15.2				13.9	–			
	10	2020–02–06	13.7	15.1				16.1	–			
	14	2020–02–10	11	13.7				12.3	–			
	17	2020–02–13	12.4	13.2				13.6	12.8			
	21	2020–02–17	12.6	13.3				12.9	13.6			
Pig 2			Left side – wound position					Right side – wound position				
	Days	Date	1	2	3	4	5	6	7	8	9	10
	0	2020–01–27	11.9	–	–	15.3	16.6	–	14.2	–	15.8	16.3
	2	2020–01–29	144	–	–	156	143	–	145	–	147	160
	4	2020–01–31	195	191	–	189	187	190	–	–	191	195
	7	2020–02–03	183	184	188	190	186	180	–	181	185	186
	10	2020–02–06	167	181	183	171	178	144	–	159	160	176
	14	2020–02–10	25.2	28.7	40.7	23.1	37.5	31.3	41	–	28.7	33.1
	17	2020–02–13	12.6	13.8	14.2	13.1	13.8	12.9	14.3	12.9	13.9	14.1
	21	2020–02–17	7.2	7.8	6.6	11	7.4	8.5	7	9	6.5	7.1

Table S3. Compilation of recorded skin surface temperature values at various control positions for both pigs during the *in vivo* wound healing cycle. The "–" sign indicates that the temperature measurements were not performed at that particular occasion.

			Left side – control position		Right side – control position	
	Days	Date	1	2	3	4
Pig 1	0	2020–01–27	–	–	–	–
	2	2020–01–29	34.2	34.4	34.6	34.6
	4	2020–01–31	34.2	33.8	33.6	34.1
	7	2020–02–03	33.7	33.6	33.6	33.4
	10	2020–02–06	33.5	33.1	33.1	33.3
	14	2020–02–10	33.5	33.1	33.6	33.2
	17	2020–02–13	33.3	33.1	33.4	32.9
	21	2020–02–17	33.6	33.4	33.6	33.3
Pig 2	0	2020–01–27	–	–	–	–
	2	2020–01–29	34.7	34.8	35.2	35.1
	4	2020–01–31	34.4	34.2	34.0	34.2
	7	2020–02–03	34.3	34.0	34.3	34.0
	10	2020–02–06	34.1	34.5	34.6	34.3
	14	2020–02–10	33.4	33.8	33.7	33.4
	17	2020–02–13	33.7	33.6	33.8	33.4
	21	2020–02–17	33.1	33.4	33.1	33.2

Table S4. Compilation of measured pH values at various wound and control positions for both pigs during the *in vivo* wound healing cycle. The "–" sign indicates that the pH measurements were not performed at that particular occasion.

			Left side – control position					Right side – control position				
	Days	Date	1	2				3				
Pig 1	0	2020–01–27	–	–				–				
	2	2020–01–29	5	5.1				5.1				
	4	2020–01–31	5.3	5.2				5.1				
	7	2020–02–03	5.3	5.5				5.3				
	10	2020–02–06	5.2	5.3				5.1				
	14	2020–02–10	5.1	5.2				5.3				
	17	2020–02–13	5.2	5.1				5.3				
	21	2020–02–17	5.2	5.1				5.3				
			Left side – wound position					Right side – wound position				
	Days	Date	1	2	3	4	5	6	7	8	9	10
	0	2020–01–27	–	–	–	–	–	–	–	–	–	–
	2	2020–01–29	8.4	–	–	7.6	8.6	–	8.6	–	8.4	8.6
	4	2020–01–31	8.2	–	8.5	8.3	–	–	–	8	8.3	8.4
	7	2020–02–03	8.1	–	–	8.2	7.8	–	–	–	8.3	–
	10	2020–02–06	7.3	–	–	7.5	7.1	–	–	7.1	6.9	6.8
	14	2020–02–10	5.3	–	5.2	–	5.3	–	–	5.4	5.3	5.2
	17	2020–02–13	5.2	–	5.3	5.2	5.2	5.4	–	–	5.4	5.3
	21	2020–02–17	5.1	–	5	5	4.9	–	–	5.4	–	5.4
			Left side – control position					Right side – control position				
	Days	Date	1					2				
Pig 2	0	2020–01–27	–					–				
	2	2020–01–29	5.3					5.3				
	4	2020–01–31	5.2					5.2				
	7	2020–02–03	5.4					5.3				
	10	2020–02–06	5.2					5.3				
	14	2020–02–10	5.3					5.5				
	17	2020–02–13	5.4					5.6				
	21	2020–02–17	5.1					5.2				
			Left side – wound position					Right side – wound position				
	Days	Date	1	2	3	4	5	6	7	8	9	10
	0	2020–01–27	–	–	–	–	–	–	–	–	–	–
	2	2020–01–29	7.8	8.3	–	–	8.5	–	–	–	8.3	8.2
	4	2020–01–31	8.2	–	8	–	8.3	–	–	8.3	8.2	7.9
	7	2020–02–03	–	–	8.4	8.6	–	–	8.3	–	8.4	–
	10	2020–02–06	7.7	7.8	–	8.1	7.8	7.8	7.5	–	–	7.2
	14	2020–02–10	5.6	5.5	5.7	–	5.8	5.6	5.5	5.5	–	5.6
	17	2020–02–13	–	5.5	–	–	5.5	–	5.7	–	5.7	–
	21	2020–02–17	5.2	5.1	–	–	5.1	–	5.5	5.4	–	5.4

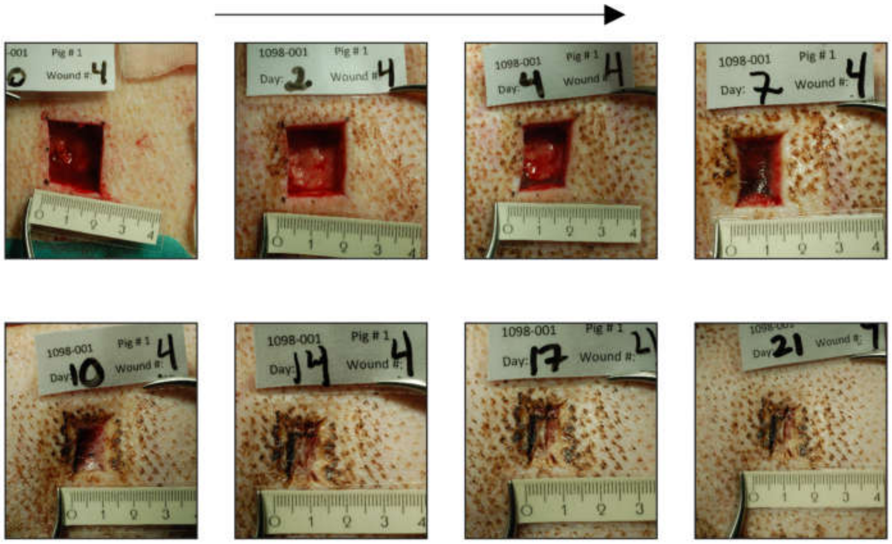

Figure S1. Representative wound images on pig 1 at various time points during the healing progression cycle. The arrow on top of the figure indicates ascending date for healing cycle and the readers are encouraged to follow the images in the arrow direction. The images presented origin from position 4 and were captured on the days when the dressing was changed. The name plate on the top of the images indicates the *in vivo* wound study number, pig number, study days when the wound dressing was changed and the wound position number. The ruler on the bottom of the images was used as a guide to set the scale when calculating the wound marked area.

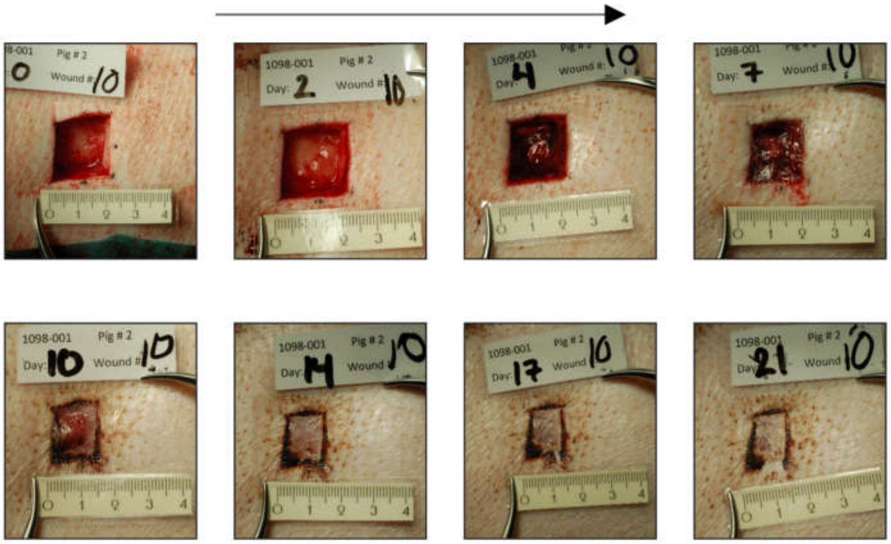

Figure S2. Representative wound images on pig 2 at various time points during the healing progression cycle. The arrow on top of the figure indicates ascending date for healing cycle and the readers are encouraged to follow the images in the arrow direction. The images presented origin from position 10 and were captured on the days when the dressing was changed. The name plate on the top of the images indicates the *in vivo* wound study number, pig number, study days when the wound dressing was changed and the wound position number. The ruler on the bottom of the images was used as a guide to set the scale when calculating the wound marked area.

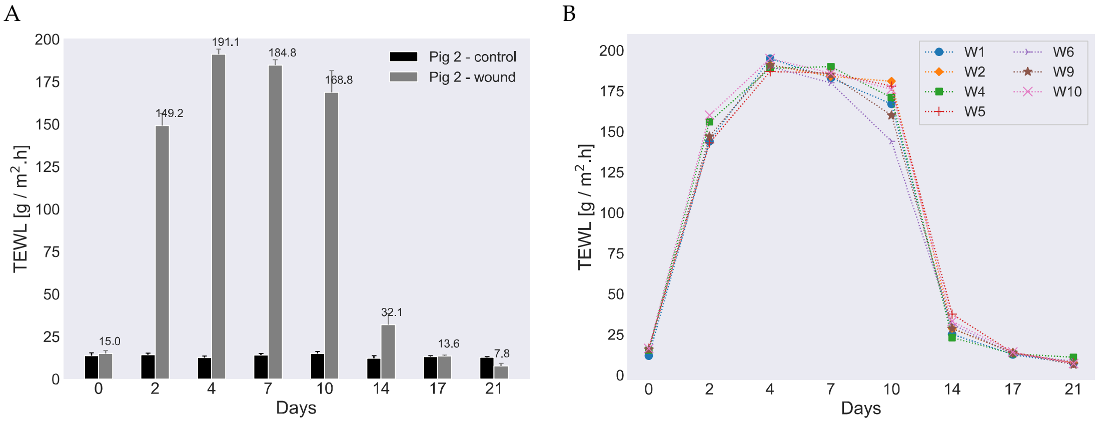

Figure S3. Variations in TEWL during the wound healing cycle. (A) Measured TEWL vs. time for control skin and healing skin for pig 2, position 1. On day 0, the readings were recorded before the wound incision. (B) TEWL values vs. time for individual wounds obtained from pig 2 when the wound dressings were changed. All the TEWL values were recorded at ambient controlled conditions, i.e. approx. 50% RH and 23 °C. Error bars depict standard deviation.

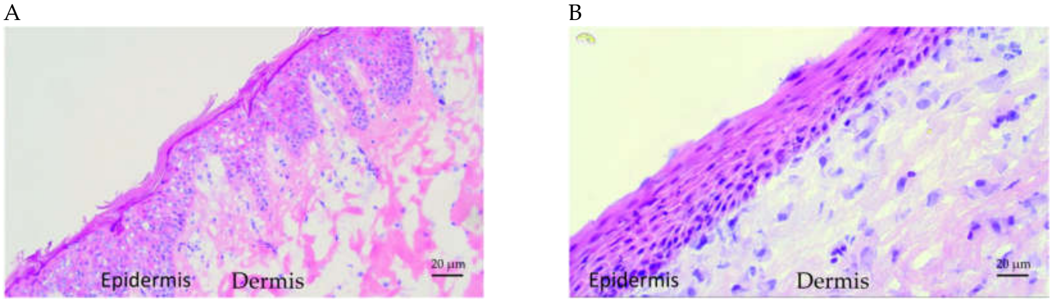

Figure S4. Classical H&E staining of control (A) and healed (B) pig skin. The skin surface is directed towards the top/left in both images. The individual epidermal layers along with dermis can be identified in the control image whereas in the healed skin, the epidermal layers are less distinguishable due to the lack of maturation.

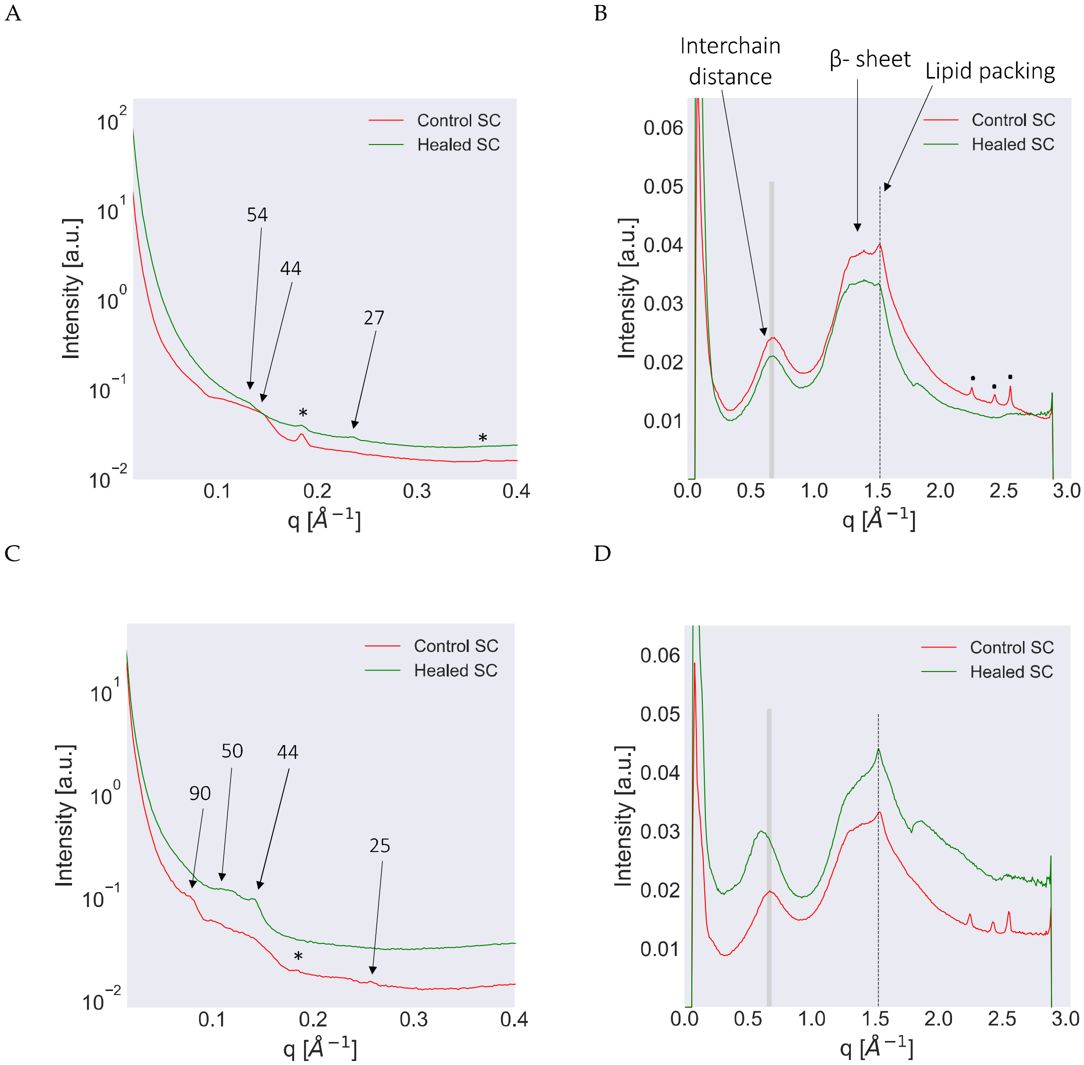

Figure S5. SWAXD spectra of control and healed SC excised from pig 2 and recorded at dry (A & B) and wet (97% RH) conditions (C & D). The SAXD spectra are provided on the left column whereas the WAXD spectra are on the right. In the SAXD region, several peaks attributed to lipid lamellar ordering and keratin packing are identified. The numbers with arrows indicate the *d*–spacing in Å for various peaks. Phase separated crystalline cholesterol is also detected in some spectra and then indicated by an asterisk (*) sign. In the WAXD region, the shaded lines indicate peaks originating from the keratin interchain distance and show a change in peak position following hydration of the sample. The lipid acyl chain ordering indicated by dotted lines do not observe a shift in peak position when comparing dry and hydrated conditions. The secondary β–sheet structure of keratin could also be detected and is marked in the plot. (•) – unknown peaks. All SWAXD measurements were performed at 32 °C which represents average physiological skin temperature.

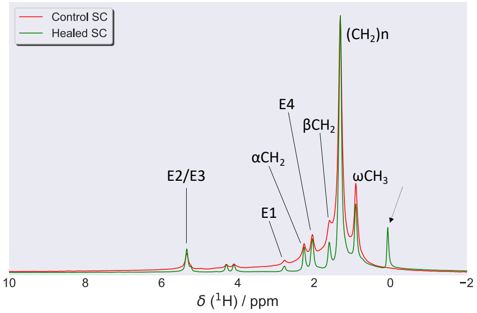

Figure S6. ^{1}H NMR measurements on control and healed SC in dry condition. All the peaks labelled in the spectra are based on the assignments provided in the main Figure 7E. Several sharp peaks attributed to various lipid molecular segments are detected in both samples, indicative of fluid lipids present in dry condition. The arrow close to 0 ppm in the healed SC is due to the silicon present in the wound dressing.

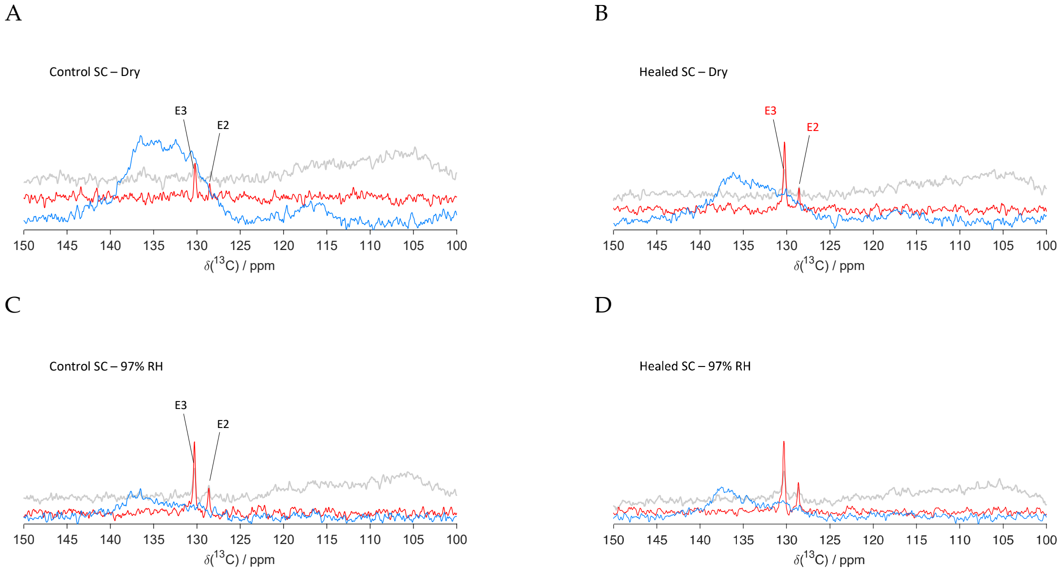

Figure S7. Natural abundance ^{13}C PTssNMR study on control (A & C) and healed SC samples (B & D) in dry (A & B) and wet (C & D) conditions for high (100 –150) ppm region. The individual DP (grey), CP (blue) and INEPT (red) spectra are overlaid in all experiments for the purpose of comparison. The resonance lines originating from lipid unsaturated double bonds are labelled in the spectra according to the assignments provided in main Figure 7E. The labelling is provided in black for control and red in healed SC when changes in the INEPT signals are observed compared to control SC.

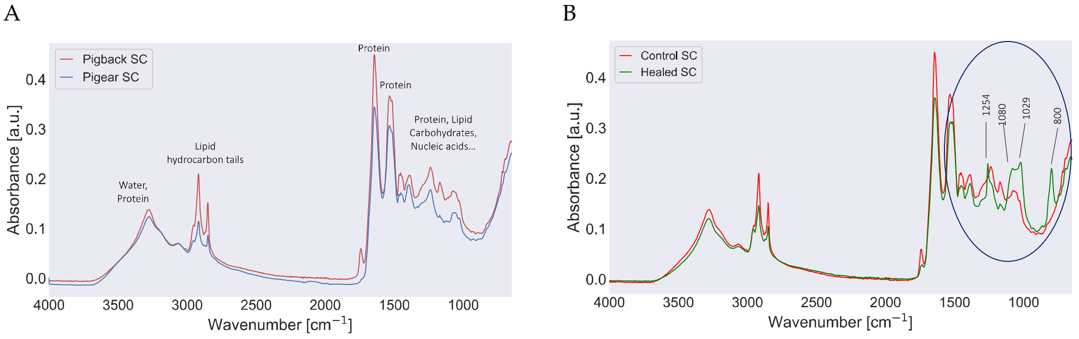

Figure S8. (A) FTIR spectra of SC in dry state, harvested from pig ear (blue) and excised skin from pigs back after terminating the *in vivo* study (red). Both SC spectra show similar trends when examining various peaks/bands originating from water, lipids, proteins etc. The difference in absorbance between the spectra is due to the amount of material in contact with ATR crystal during measurements. (B) FTIR spectra control (red) and healed (green) SC for pig 2 harvested after the *in vivo* study. In the region between 800 and 1500 cm^{-1} (marked with circle), differences are observed between control and healed SC. The contour at ca. 1080 cm^{-1} may be attributed to the PO_2^- stretch of lipids and likely originates from the phospholipid, and the peak at 1254 cm^{-1} is contributed due to amide III band. The peak at 1029 cm^{-1} might be C–H deformation of carbohydrates, an evidence for the presence of glycosphingolipids in the healed sample. The band close to 800 cm^{-1} is due to the silicon contamination from the wound dressing.
